# FBP1‐Mediated Compartmentalization of Glycolytic Enzyme Complexes Suppresses PKM2 Function to Ameliorate MASLD Progression

**DOI:** 10.1002/advs.77875

**Published:** 2026-09-27

**Authors:** Busong Wang, Yiping Tang, Na Li, Xuening Dai, Jing Wu, Bin Xue, Yuxin Chen, Jingzi Zhang, Lingdong Kong, Chaojun Li, Lei Fang

**Affiliations:** ^1^ State Key Laboratory of Pharmaceutical Biotechnology Institute of Chinese Medicine Department of Laboratory Medicine Nanjing Drum Tower Hospital Affiliated Hospital of Medical School Medical School of Nanjing University Nanjing People's Republic of China; ^2^ State Key Laboratory of Pharmaceutical Biotechnology School of Life Sciences Nanjing University Nanjing People's Republic of China; ^3^ Chemistry and Biomedicine Innovation Center Jiangsu Key Laboratory of Molecular Medicine Medical School of Nanjing University Nanjing People's Republic of China; ^4^ State Key Laboratory of Reproductive Medicine and Offspring Health Nanjing Medical University Nanjing People's Republic of China

**Keywords:** fructose‐1,6‐bisphosphatase 1 (fbp1), glycolytic enzyme compartmentalization, metabolic dysfunction‐associated steatohepatitis (mash), metabolic dysfunction‐associated steatotic liver disease (masld), pyruvate kinase m2 (pkm2)

## Abstract

Metabolic dysfunction‐associated steatotic liver disease (MASLD) is driven by aberrant hepatocyte glycolytic reprogramming. Fructose‐1,6‐bisphosphatase 1 (FBP1), a key gluconeogenic enzyme, has poorly defined roles in MASLD progression. Here, we show that FBP1 is consistently downregulated during MASLD progression, with a progressive decline from MASL to MASH in clinical specimens and key preclinical models. Mechanistically, FBP1 directly interacts with glycolytic enzymes Pyruvate kinase M2 (PKM2), Enolase 1 (ENO1), and Lactate Dehydrogenase A (LDHA) to form compartmentalized complexes, suppressing PKM2 activity and restricting excessive glycolysis and lipid accumulation in hepatocytes. Domain mapping defined the precise interaction interface between FBP1 189–235 aa and PKM2 390–531 aa, and disruption of this binding abrogated FBP1's glycolysis‐repressive effects in hepatocytes. In vivo, only liver‐specific overexpression of enzymatically inactive but binding‐competent FBP1 G260R, not its interaction‐deficient mutant FBP1 G260R‐Δ189‐235, alleviated hepatic steatosis, inflammation, and fibrosis in diet‐induced MASLD mice. This study reveals a non‐canonical function of FBP1 independent of its enzymatic activity in regulating glycolytic complex assembly and identifies the FBP1‐PKM2 interaction as a novel candidate intervention target for MASLD.

## Graphical abstract

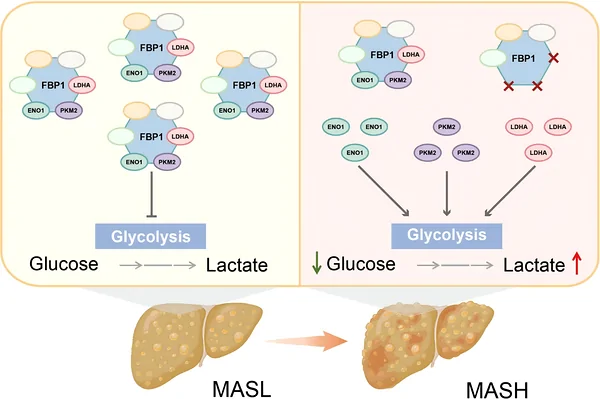

**Abbreviations**
AAV: adeno‐associated virus
CD: control diet
CDAHFD: choline‐deficientamino‐acid‐defined, high‐fat diet
Co‐IP: Co‐immunoprecipitation
DHAP: Dihydroxyacetone phosphate
ENO1: enolase 1
F6P: fructose‐6‐phosphate
FBP: Fructose‐1,6‐bisphosphate
FBP1: fructose‐1,6‐bisphosphatase 1
FRAP: fluorescence recovery after photobleaching
GA3P: Glyceraldehyde‐3‐phosphateG6P, Glucose‐6‐phosphate
GST: glutathione S‐transferase
H&E: hematoxylin and eosin
HFHCD: high‐fat, high‐cholesterol diet
HSCs: hepatic stellate cells
IF: Immunofluorescence
IHC: Immunohistochemistry
LDHA: lactate dehydrogenase A
MASH: metabolic dysfunction‐associated steatohepatitis
MASLD: metabolic dysfunction‐associated steatotic liver disease
NAS: NAFLD Activity Score
OA: oleic acid
PA: palmitic acid
PKM2: pyruvate kinase M2
PPI: protein‐protein interaction
qPCR: quantitative real‐time polymerase chain reaction
SAF: steatosis, activity, fibrosis
SEM: standard error of the mean
TurboID: TurboID proximity labeling enzyme
WB: Western blot
WD: Western diet
WT: wild‐type
α‐SMA: alpha‐smooth muscle actin

## 1 Introduction

Metabolic dysfunction‐associated steatotic liver disease (MASLD), formerly known as metabolic dysfunction‐associated fatty liver (MAFLD) and non‐alcoholic fatty liver (NAFLD) [1, 2], has become the most prevalent chronic liver disease worldwide, affecting more than 25% of the global population [3]. The MASLD spectrum includes simple steatosis (metabolic dysfunction‐associated steatotic liver, MASL), metabolic dysfunction‐associated steatohepatitis (MASH), and ultimately progresses to liver fibrosis, cirrhosis, and hepatocellular carcinoma (HCC) [4, 5]. Unlike advanced fibrosis and cirrhosis, the MASH stage remains partially reversible and can regress to MASL with appropriate interventions, making early diagnosis and intervention at the MASH stage of paramount clinical importance [6]. However, the molecular mechanisms underlying MASLD progression remain incompletely understood, and therapeutic options remain limited, and approved agents are restricted to selected patient populations [7, 8].

Aberrant metabolic reprogramming of hepatocytes, particularly excessive activation of the glycolytic pathway, is a critical factor driving MASLD pathogenesis [9, 10]. Overactivation of glycolysis promotes multiple pathological processes in the liver, including lipid synthesis, immune inflammation activation, hepatic stellate cell fibrosis, and carcinogenesis [11]. Therapeutic strategies targeting the glycolytic pathway to regulate glycolytic flux have been shown to significantly alleviate MASLD progression [12]. Inhibiting key glycolytic enzymes to restore the homeostatic balance between hepatic glycolysis and gluconeogenesis represents an important direction for the development of novel MASLD therapies [13].

Fructose‐1,6‐bisphosphatase 1 (FBP1) is a rate‐limiting gluconeogenic enzyme with both catalytic and non‐enzymatic functions, including regulation of insulin signaling, glycolysis, and tumorigenesis [14, 15, 16, 17, 18]. FBP1 acts as a scaffold to form protein complexes to accelerate AKT serine/threonine kinase (AKT) dephosphorylation, thereby terminating insulin signaling [14]; as a direct transcriptional target of p53, FBP1 inhibits the transition of senescent hepatocytes from MASH to HCC by suppressing AKT activity [16]; FBP1 directly binds to the inhibitory domain of hypoxia‐inducible factor 1α (HIF‐1α) to suppress its transcriptional activity, thereby blocking hypoxia‐induced glycolysis and angiogenesis in clear cell renal cell carcinoma [19]. Furthermore, FBP1 exerts phosphatase activity to suppress the progression of various malignancies [20, 21, 22]. Despite extensive research on FBP1 biological functions, the role of FBP1 in MASLD progression remains unclear.

Pyruvate kinase M2 (PKM2), a terminal glycolytic enzyme frequently upregulated in MASLD, drives MASLD progression via metabolic reprogramming and transcriptional co‐activation [23, 24, 25]. Multiple studies have reported that PKM2 expression is significantly upregulated during MASLD progression [26, 27], and reducing PKM2 expression or enzymatic activity markedly ameliorates diet‐induced liver inflammation and fibrosis phenotypes in mice [28, 29, 30, 31]. Recent studies have found that key glycolytic enzymes can assemble into dynamically regulated compartmentalized complexes through specific protein‐protein interactions, enriching the catalytic enzymes of each step of glycolysis in close proximity, reducing the diffusion loss of intermediate metabolites, and significantly improving the overall catalytic efficiency of glycolysis [32, 33, 34, 35]. For example, glycolytic enzyme compartmentalized complexes can enhance tumor glycolytic energy supply and promote cancer cell metastasis [36]. However, whether gluconeogenic enzymes regulate these complexes in MASLD is unknown.

Herein, utilizing validated diet‐induced MASLD mouse models, clinical liver specimens, in vitro hepatocyte systems, and multiple molecular approaches, we demonstrate that FBP1 is progressively downregulated during the MASL‐to‐MASH transition. Proximity labeling combined with quantitative proteomics confirmed direct binding of FBP1 to core glycolytic enzymes PKM2, Enolase 1 (ENO1), and Lactate Dehydrogenase A (LDHA). FBP1 directly interacts with PKM2, ENO1, and LDHA to form dynamic glycolytic complexes, suppressing PKM2 activity and glycolysis in a catalytic‐independent manner. Disruption of the FBP1‐PKM2 interaction abrogates FBP1's protective effects against hepatic steatosis, inflammation, and fibrosis in vivo. Overall, this study reveals a novel non‐catalytic regulatory role of FBP1 in glycolytic assembly, clarifies its underlying mechanism in disease deterioration, and presents FBP1 as a promising candidate intervention target for MASLD.

## 2 Materials and Methods

Please see the Supporting Information for detailed materials and methods.

## 3 Results

### 3.1 FBP1 is Consistently Downregulated During MASL‐to‐MASH Transition in Multiple Preclinical and Clinical Models

**FIGURE 1:**
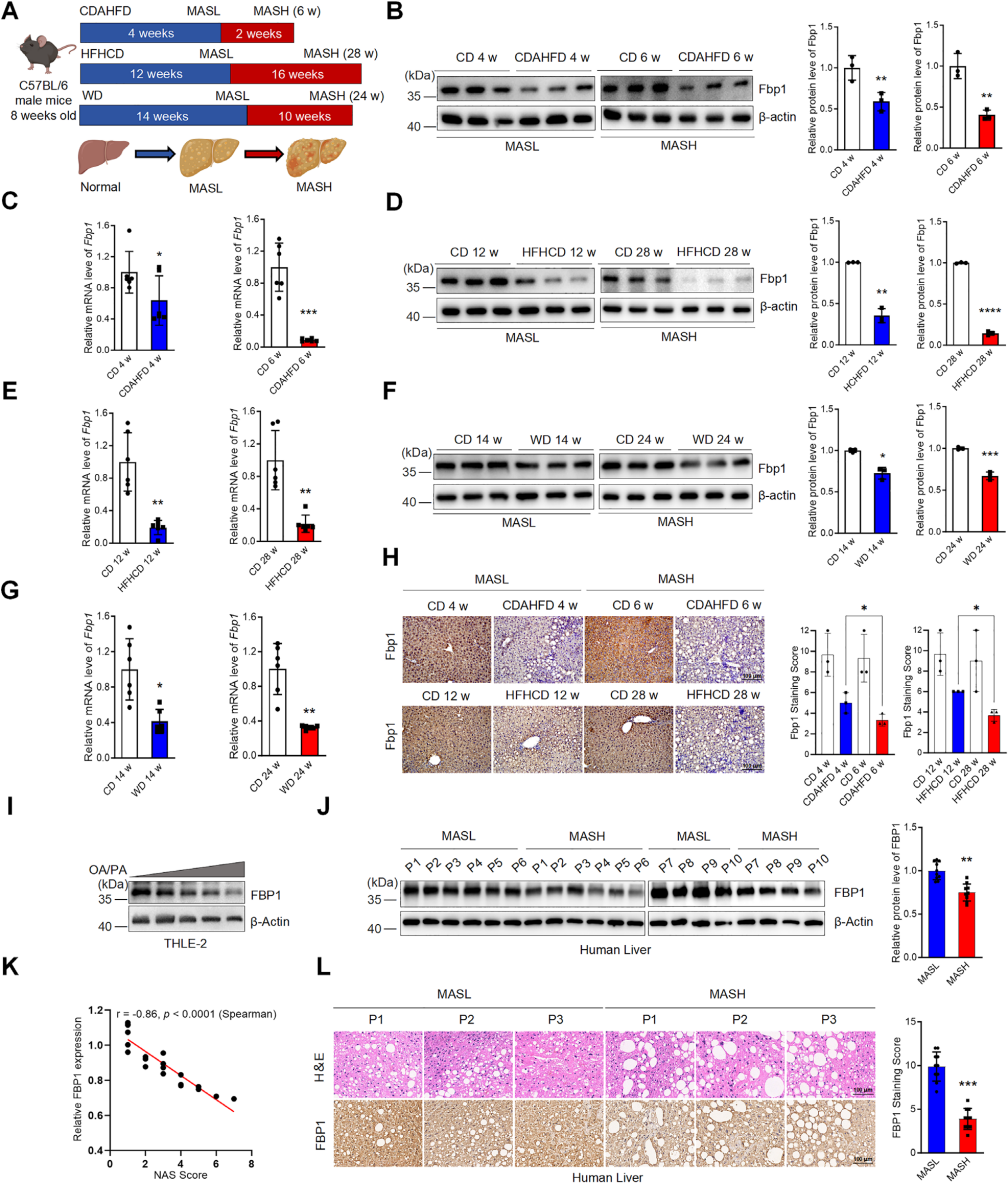
FBP1 expression is progressively downregulated during MASLD progression in human and mouse livers. (A) Schematic of three classic MASLD mouse models: CDAHFD, HFHCD, and WD. (B, D, F) WB analysis of Fbp1 protein in liver tissues from CDAHFD (B), HFHCD (D), and WD (F) mice at MASL and MASH stages; CD as control (*n* = 3 per group). (C, E, G) qPCR analysis of *Fbp1* mRNA in liver tissues from the above models at indicated time points (*n* = 6 per group). (H) IHC staining of Fbp1 in CDAHFD and HFHCD mouse livers at MASL and MASH stages and quantitative analysis of IHC scores (*n* = 3 per group). Scale bars: 100 µm. (I) WB analysis of FBP1 protein in THLE‐2 cells treated with gradient OA/PA for 24 h. (J) WB analysis of FBP1 protein in liver tissues from MASL and MASH patients, with a quantitative bar graph of relative FBP1 protein levels (*n* = 10 per group). (K) Spearman correlation between FBP1 expression and NAS score (*n* = 20, r = −0.86, *p* < 0.00001). (L) Representative H&E and FBP1 IHC staining of liver tissues from MASL and MASH patients, with a quantitative bar graph of IHC scores (*n* = 10 per group). Scale bars: 100 µm. β‐Actin served as a loading control for all WB assays. Data are presented as mean ± SEM. Significance was assessed by one‐way ANOVA with Tukey's test for (B, D, F, H), two‐sided unpaired Student's *t*‐test for (C, E, G, J), Mann‐Whitney U test for IHC IRS scoring in (L), or Spearman's rank correlation test for (K). ^*^ *p* < 0.05, ^**^ *p* < 0.01, ^***^ *p* < 0.001, ^****^ *p* < 0.0001 vs. control group.

To investigate the changes in FBP1 expression levels during MASLD progression, we established three diet‐induced mouse models recapitulating the MASL‐to‐MASH transition: C57BL/6 mice were fed a choline‐deficient, amino‐acid‐defined, high‐fat diet (CDAHFD) for 4 or 6 weeks, a high‐fat, high‐cholesterol diet (HFHCD) for 12 or 28 weeks, or a Western diet (WD) for 14 or 24 weeks, respectively (Figure 1A) [37, 38, 39]. All of which recapitulated the MASL‐to‐MASH transition (Figure S1A–E).

Western blot (WB) and quantitative real‐time PCR (qPCR) analyses of liver tissues showed that Fbp1 protein and mRNA levels progressively decreased from the MASL stage to the MASH stage in the CDAHFD and HFHCD models. In the WD model, Fbp1 was markedly downregulated at the MASL stage (14 weeks) and remained persistently low at the MASH stage (24 weeks), with no significant further reduction. These changes were accompanied by upregulation of glycolytic enzymes Hk2 and Pkm2 across all three models (Figure 1B–G and Figure S1F). Immunohistochemical (IHC) staining of mouse liver further confirmed reduced Fbp1 immunoreactivity in MASL and near‐complete loss in MASH livers (Figure 1H). We then investigated the changes in FBP1 expression in an in vitro MASLD cell model by treating THLE‐2 cells with gradient concentrations of oleic acid (OA)/palmitic acid (PA) to induce hepatocyte lipid accumulation. Western blot results showed that FBP1 protein expression decreased in a dose‐dependent manner with increasing OA/PA concentrations (Figure 1I), indicating that excess free fatty acids directly inhibit FBP1 expression in hepatocytes.

Then we determined the changes in FBP1 expression in clinical patient samples of MASL and MASH. Analysis of the Human Protein Atlas (HPA) database (https://www.proteinatlas.org/) revealed reduced *FBP1* mRNA in MASH livers (Figure S1G). We further analyzed 20 biopsy‐proven MASLD patients (1:1 matched MASL/MASH, Table S1). Western blot analysis with grayscale quantification showed that FBP1 protein expression levels were markedly decreased in MASH liver tissues compared with MASL liver tissues (Figure 1J), and Spearman rank correlation analysis demonstrated a significant negative correlation between relative FBP1 expression and NAFLD Activity Score (NAS) in this cohort (Figure 1K). IHC staining with IRS scoring confirmed that FBP1 protein expression was markedly reduced in MASH patient livers compared with MASL controls, correlating with the increased steatosis severity shown by H&E staining (Figure 1L). These results indicate that hepatic FBP1 expression is significantly suppressed in MASH patients compared with MASL patients. Hepatocyte nuclear factor 4α (HNF4α), as a core transcriptional regulator of *FBP1* [40, 41], was selected as the candidate upstream molecule, and we examined its expression and subcellular localization during MASLD progression. Total hepatic Hnf4α protein levels progressively decreased in a disease stage‐dependent manner (Figure S1H), and nuclear‐cytoplasmic fractionation further confirmed a significant reduction of nuclear Hnf4α abundance in MASH‐stage livers with no significant change in cytosolic Hnf4α levels (Figure S1I). These results indicate that reduced nuclear localization and transcriptional activity of HNF4α mediate the transcriptional downregulation of *FBP1* during MASLD development.

Collectively, these results demonstrate that hepatic FBP1 expression is consistently suppressed during MASLD progression across all preclinical models, cell systems, and clinical samples. The CDAHFD and HFHCD models recapitulate a progressive decline along the MASL‐to‐MASH transition, while the WD model shows early downregulation followed by sustained low expression throughout disease advancement.

### 3.2 Mapping Comprehensive FBP1‐Interacting Protein Network via TurboID Proximity Labeling Coupled With Mass Spectrometry

**FIGURE 2:**
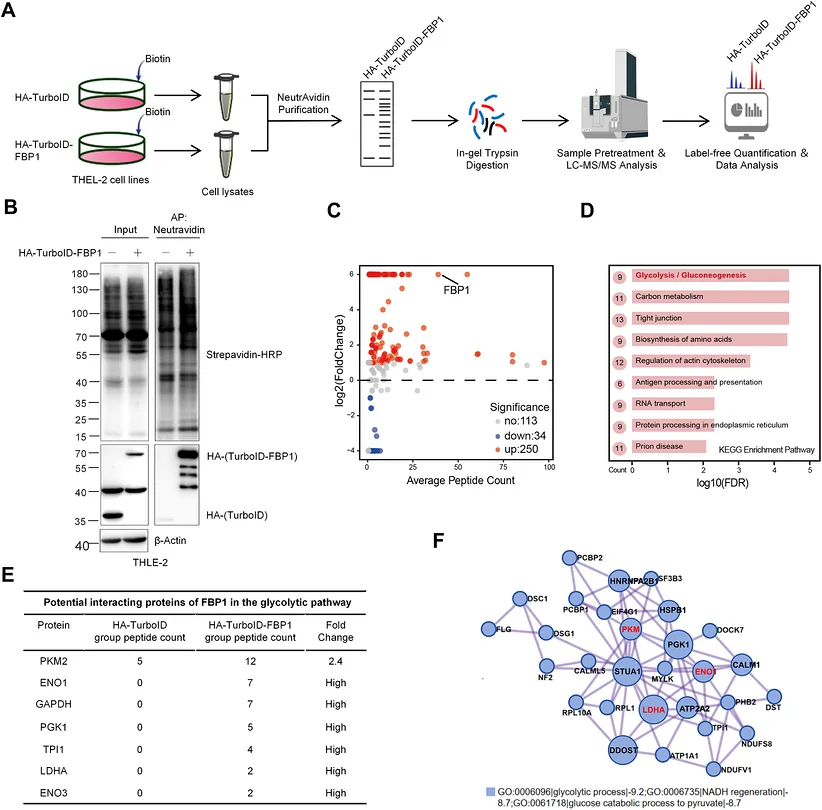
Mapping Comprehensive FBP1‐Interacting Protein Network via TurboID Proximity Labeling Coupled with Mass Spectrometry. (A) Schematic of TurboID‐based proximity labeling workflow in THLE‐2 cells. (B) WB validation of biotinylated proteins and HA‐TurboID‐FBP1 expression. (C) Scatter plot of differentially interacting proteins. (D) KEGG enrichment analysis of FBP1 interactors. (E) List of key glycolytic proteins identified as potential FBP1 interactors, with peptide counts and fold changes relative to HA‐TurboID control. (F) Protein‐protein interaction network of FBP1 and glycolytic enzymes. β‐Actin served as a loading control.

To further explore the molecular mechanisms underlying FBP1 function in hepatocytes, we used TurboID‐based proximity labeling technology combined with label‐free quantitative mass spectrometry (MS) to identify FBP1‐interacting proteins in THLE‐2 cells (Figure 2A). Western blot results confirmed the successful expression of HA‐TurboID and HA‐TurboID‐FBP1 in THLE‐2 cells and their ability to catalyze intracellular protein biotinylation. Furthermore, NeutrAvidin successfully enriched TurboID‐fused FBP1 and its catalyzed biotinylated proteins (Figure 2B).

We identified 250 potential FBP1 interactors (Figure 2C). Kyoto Encyclopedia of Genes and Genomes (KEGG) enrichment analysis of the identified interacting proteins revealed that proteins associated with the glycolytic process were significantly enriched among FBP1‐interacting proteins (Figure 2D), along with other biological processes such as carbon metabolism, tight junction, and biosynthesis of amino acids. Further inspection of the glycolytic enzyme profile showed that hexokinase 2 (HK2) and key enzymes of the pentose phosphate pathway (PPP) were not enriched as high‐confidence proximal proteins. Instead, FBP1 was found in close proximity to a panel of glycolytic enzymes acting downstream of HK2, including Glyceraldehyde‐3‐phosphate dehydrogenase (GAPDH), PKM2, ENO1, Phosphoglycerate kinase 1 (PGK1), Triosephosphate isomerase 1 (TPI1), LDHA, and Enolase 3 (ENO3) (Figure 2E). The peptide counts of these proteins, especially the key glycolytic enzyme PKM2, were significantly increased in the HA‐TurboID‐FBP1 group compared with the HA‐TurboID control group, indicating their specific enrichment (Figure 2E). Protein‐protein interaction (PPI) network analysis was constructed to illustrate the candidate interaction relationships between FBP1 and all identified proteins, including glycolytic enzymes (Figure 2F). Key glycolytic proteins such as PKM2, ENO1, and LDHA were located at the core of the network, suggesting an important role of FBP1 in regulating glycolytic reactions in hepatocytes. Notably, PKM2 exhibited the highest enrichment and most stable interaction among all identified glycolytic enzymes. As the terminal rate‐limiting enzyme of the glycolytic pathway that determines overall pathway flux output, PKM2 was selected as the core representative candidate for subsequent in‐depth mechanistic dissection. MS/MS spectra of these proteins confirmed the specificity of our proteomic data (Figure S2A–C).

Collectively, these data indicate that FBP1 forms complexes with multiple glycolytic enzymes in hepatocytes, suggesting a role in glycolysis regulation beyond gluconeogenesis.

### 3.3 FBP1 Forms Compartmentalized Complexes With Key Glycolytic Enzymes in Hepatocytes

**FIGURE 3:**
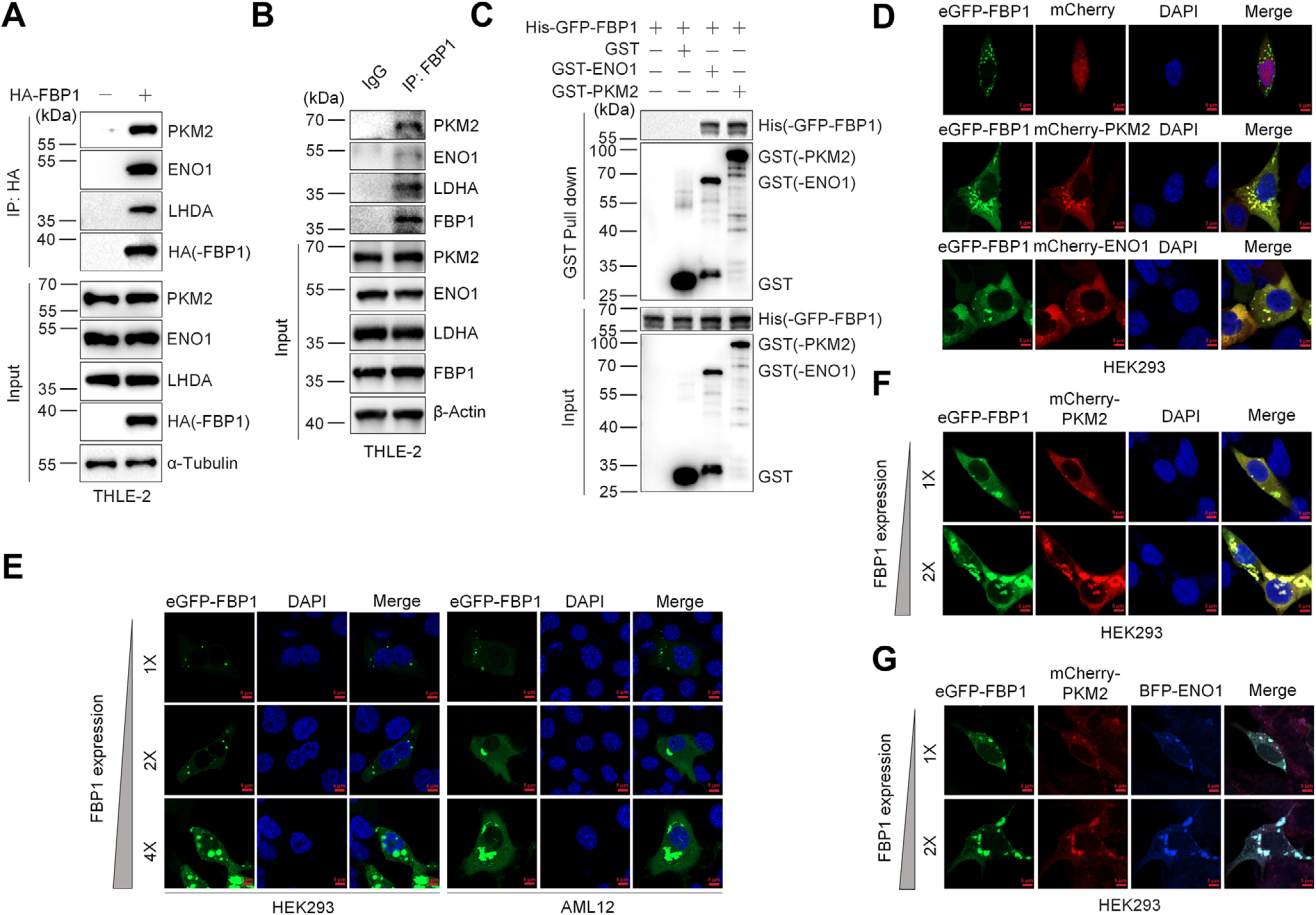
FBP1 Forms Compartmentalized Complexes with Key Glycolytic Enzymes in Hepatocytes. (A) Co‐immunoprecipitation (Co‐IP) assay in HA‐FBP1‐transfected THLE‐2 cells. Immunoprecipitation was performed with an anti‐HA antibody, and precipitated proteins were detected with anti‐PKM2, anti‐ENO1, anti‐LDHA, and anti‐HA antibodies. (B) Endogenous Co‐IP assay in THLE‐2 cells, with anti‐FBP1 antibody for immunoprecipitation and normal IgG as a negative control. (C) In vitro GST pull‐down assay. Recombinant His‐GFP‐FBP1 was incubated with GST, GST‐PKM2, or GST‐ENO1 protein, respectively. (D) Immunofluorescence co‐localization of eGFP‐FBP1 with mCherry, mCherry‐PKM2, or mCherry‐ENO1 in HEK293 cells. Scale bars: 5 µm. (E) Dose‐dependent formation of eGFP‐FBP1 cytoplasmic puncta at different expression levels in HEK293 cells and AML12 cells transfected with 1, 2, or 4 µg eGFP‐FBP1 plasmid. Scale bars: 5 µm. (F) Dose‐dependent co‐localization of eGFP‐FBP1 and mCherry‐PKM2 in HEK293 cells transfected with 2 or 4 µg eGFP‐FBP1 plasmid. Scale bars: 5 µm. (G) Dose‐dependent triple immunofluorescence co‐localization of eGFP‐FBP1, mCherry‐PKM2, and BFP‐ENO1 in HEK293 cells transfected with 2 or 4 µg eGFP‐FBP1 plasmid. Scale bars: 5 µm. Nuclei were stained with DAPI for all immunofluorescence assays.

To validate the interactions between FBP1 and the glycolytic enzymes identified by mass spectrometry (PKM2, ENO1, LDHA), we first performed co‐immunoprecipitation (co‐IP) experiments in THLE‐2 cells expressing exogenous FBP1. Immunoprecipitation results confirmed that exogenous FBP1 interacted with PKM2, ENO1, and LDHA in THLE‐2 cells (Figure 3A and Figure S3A). Next, we validated the interaction between endogenous FBP1 and glycolytic enzymes in THLE‐2 cells. Immunoprecipitation with an anti‐FBP1 antibody clearly pulled down endogenous PKM2, ENO1, and LDHA (Figure 3B), indicating that FBP1 forms complexes with these enzymes under physiological conditions in hepatocytes. To determine whether FBP1 directly interacts with glycolytic enzymes, we performed in vitro GST pull‐down assays using recombinant proteins. Incubation of His‐GFP‐FBP1 with GST, GST‐ENO1, or GST‐PKM2 showed that His‐GFP‐FBP1 was pulled down by GST‐ENO1 and GST‐PKM2, but not by the GST control (Figure 3C), confirming direct physical binding between FBP1 and these two glycolytic enzymes.

We further validated the interaction between FBP1 and glycolytic enzymes in cells by immunofluorescence staining. Immunofluorescence results showed that eGFP‐FBP1 colocalized with mCherry‐PKM2 or mCherry‐ENO1 (red) in cytoplasmic compartmentalized complexes, as evidenced by the yellow signal in the merged images (Figure 3D). Surprisingly, overexpressed eGFP‐FBP1 (green) spontaneously formed punctate aggregates in the cytoplasm of HEK293 and AML12 cells, and the size and number of these puncta increased in a dose‐dependent manner with increasing intracellular FBP1 expression levels (Figure 3E and Figure S3B). Quantitative analysis confirmed a dose‐dependent elevation in the FBP1 puncta area fraction in both cell lines (Figure S3C). This phenomenon was also observed in HepG2 cells (Figure S3D), suggesting that FBP1 forms compartmentalized complexes in both hepatocytes and tumor cells. Consistently, endogenous immunofluorescence imaging revealed that endogenous Fbp1 presents fine punctate aggregates on a diffuse cytoplasmic background in normal mouse liver tissues and AML12 normal hepatocytes, while such aggregation is markedly diminished in liver tissues from MASH mice (Figure S3E, F), further confirming the genuine existence of FBP1‐mediated compartmentalized complexes under physiological conditions. In contrast, no such compartmentalization was observed when PKM2 expression was increased in a gradient manner (Figure S3G, H). Furthermore, the colocalization signal between mCherry‐PKM2 and eGFP‐FBP1 compartmentalized complexes was significantly enhanced with increasing FBP1 expression levels (Figure 3F). In addition, triple transfection experiments co‐expressing eGFP‐FBP1, mCherry‐PKM2, and BFP‐ENO1 further demonstrated that FBP1 can colocalize with both glycolytic enzymes simultaneously (Figure 3G), indicating that the interaction between FBP1 and glycolytic enzymes such as PKM2 and ENO1 in cells is dependent on FBP1 abundance.

To characterize the biophysical properties of the FBP1 compartmentalized puncta observed in cells, we first analyzed the dynamic exchange capacity of their internal molecules using fluorescence recovery after photobleaching (FRAP) technology. The results showed that the fluorescence signal in the bleached area gradually recovered over time after photobleaching, and quantitative analysis indicated that the final exchangeable fraction was approximately 60% in HEK293 cells expressing eGFP‐FBP1, suggesting that the FBP1‐mediated compartmentalized complex structure is not a static protein aggregate, and the FBP1 molecules inside can undergo continuous dynamic exchange with free FBP1 molecules in the surrounding cytoplasm (Figure S3I). To further explore the molecular interaction basis driving the formation of FBP1 compartmentalized complexes, we treated HEK293 cells expressing eGFP‐FBP1 with 1,6‐hexanediol, which specifically disrupts weak hydrophobic interactions between proteins. Treatment with 1,6‐hexanediol disrupted these complexes, indicating they are formed via weak hydrophobic interactions (Figure S3J).

In addition, nuclear‐cytoplasmic fractionation assays in AML12 cells showed that FBP1 overexpression markedly reduces the nuclear abundance of PKM2, indicating that FBP1‐mediated compartmentalization not only sequesters PKM2 in the cytoplasm but also restricts its nuclear translocation (Figure S3K).

Taken together, these results demonstrate that FBP1 drives the assembly of dynamic glycolytic compartmentalized complexes in a dose‐dependent manner, and these complexes possess dynamic molecular exchange properties.

### 3.4 FBP1 Directly Interacts With the C‐terminal Catalytic Domain of PKM2 via its 189–235 aa Domain

**FIGURE 4:**
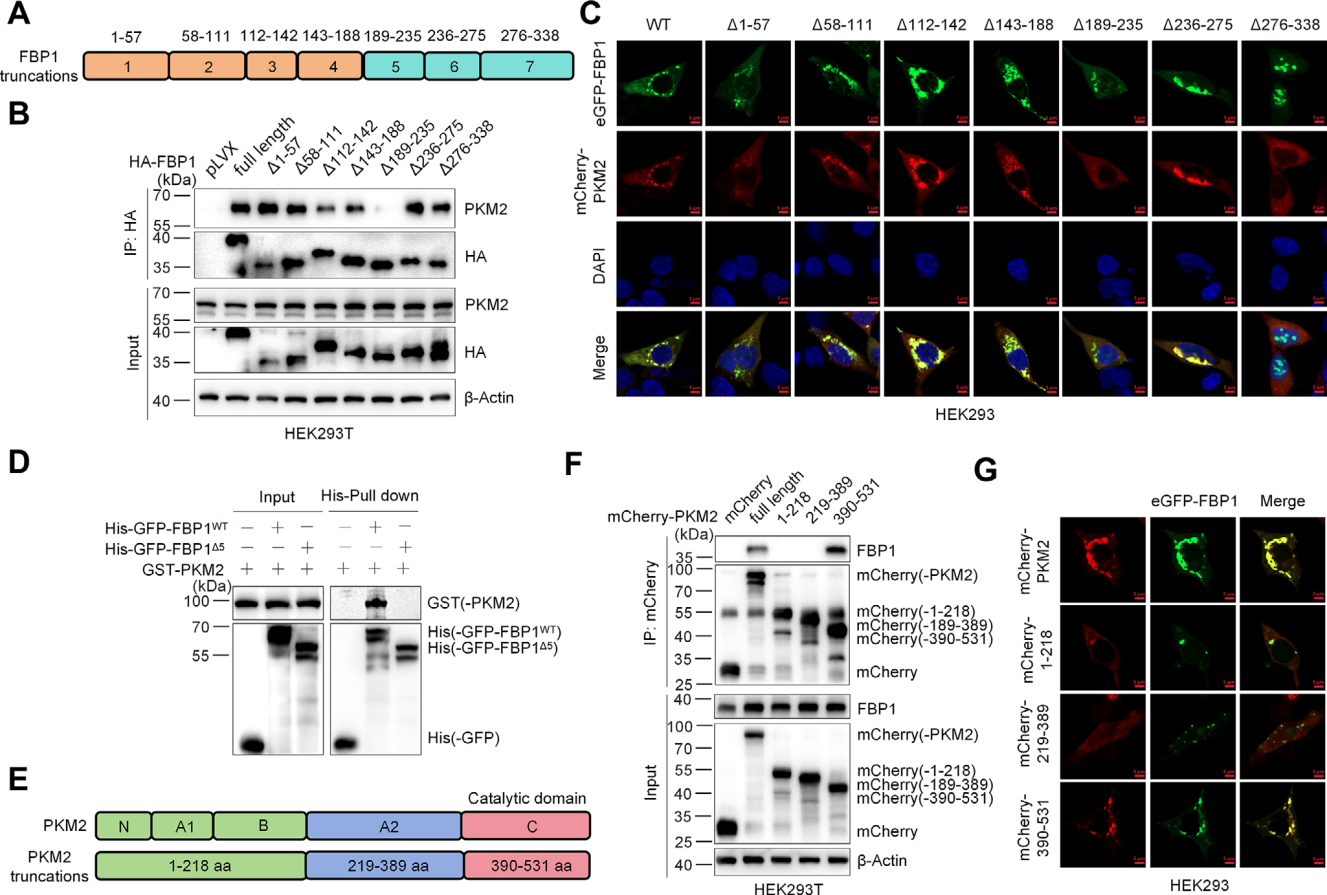
FBP1 Directly Interacts with the C‐terminal Catalytic Domain of PKM2 via Its 189–235 aa Domain. (A) Schematic of human FBP1 domain structure. (B) Co‐IP to map the PKM2‐binding domain of FBP1 (HEK293T cells co‐transfected with HA‐tagged full‐length FBP1 or truncations and PKM2). (C) Co‐localization analysis of eGFP‐tagged FBP1 truncations with mCherry‐PKM2 in HEK293 cells (scale bars: 5 µm). (D) In vitro His pull‐down confirming that FBP1 Δ5 (189‐235 aa deletion) abolishes FBP1‐PKM2 interaction. (E) Schematic of human PKM2 domain structure. (F) Reverse co‐IP to map the FBP1‐binding domain of PKM2 (HEK293T cells co‐transfected with FBP1 and mCherry‐tagged full‐length PKM2 or truncations). (G) Co‐localization analysis of eGFP‐FBP1 with mCherry‐tagged full‐length PKM2 and its truncations in HEK293 cells. Scale bars: 5 µm. β‐Actin served as the loading control for all Western blot assays. Nuclei were stained with DAPI for all immunofluorescence assays.

To elucidate the molecular basis of the interaction between FBP1 and PKM2, we performed systematic domain truncation experiments to map the key regions mediating their binding. First, we constructed a series of FBP1 deletion mutants based on the domain architecture of FBP1 (Figure 4A) [19]. Co‐IP experiments in HEK293T cells showed that full‐length FBP1 and its Δ1‐57, Δ58‐111, Δ112‐142, Δ143‐188, Δ236‐275, and Δ276‐338 mutants all effectively co‐precipitated endogenous PKM2; in contrast, the Δ189‐235 mutant (Δ5 mutant) failed to interact with PKM2, indicating that the 189–235 aa domain of FBP1 is essential for binding to PKM2 (Figure 4B). Immunofluorescence confirmed reduced colocalization between FBP1 Δ189‐235 and PKM2 (Figure 4C). Notably, the Δ276‐338 mutant showed reduced cytoplasmic complex formation, suggesting this region is required for compartmentalized complex assembly (Figure 4C). To validate the direct interaction between the 189 and 235 aa domain of FBP1 and PKM2, we performed in vitro His pull‐down assays using recombinant His‐GFP‐FBP1 wild‐type and 189–235 aa domain deletion mutant. In vitro pull‐down assays verified that the 189–235 aa domain mediates direct FBP1‐PKM2 binding (Figure 4D).

Next, we investigated the key region of PKM2 that interacts with FBP1. PKM2 consists of an N‐terminal regulatory region (including N, A1, and B domains, 1–218 aa), an intermediate domain (A2, 219–389 aa), and a C‐terminal catalytic domain (C, 390–531 aa) that contains the critical tetramerization interface and allosteric binding sites for its activator fructose‐1,6‐bisphosphate (FBP) [42, 43, 44] (Figure 4E). We constructed three PKM2 truncation mutants (1‐218, 219–389, 390–531) for co‐IP experiments. The results showed that full‐length PKM2 and the C‐terminal catalytic domain (390‐531 aa) interacted with endogenous FBP1, while the 1–218 aa and 219–389 aa domains failed to bind FBP1 (Figure 4F). Immunofluorescence staining further confirmed that the 390–531 aa domain of mCherry‐PKM2 exhibited extensive colocalization with eGFP‐FBP1 in the cytoplasm (Figure 4G).

To further investigate the structural basis of the interaction between FBP1 and PKM2, molecular docking simulations were performed between FBP1 and PKM2. HADDOCK molecular docking (score: −73.1±5.5) revealed that FBP1 binds to PKM2's active site (Lys505) and tetramerization interface (Val527, Pro529, Pro531) (Figure S4A, B). These structural findings suggest that FBP1 may inhibit the enzymatic activity of PKM2 by binding to its residues 390–531 domain.

Collectively, these data demonstrate that the 189–235 aa domain of FBP1 directly interacts with the C‐terminal catalytic domain (390‐531 aa) of PKM2, providing a key structural basis for the assembly of FBP1‐driven glycolytic compartmentalized complexes.

### 3.5 FBP1 Suppresses Glycolysis and Lipid Accumulation by Inhibiting PKM Activity via Direct Interaction

Building on our previous finding that FBP1 directly binds to key glycolytic enzymes and mediates the assembly of compartmentalized complexes, we further investigated the regulatory roles of this protein interaction in glycolytic pathway activity and the pathogenesis of MASLD.

**FIGURE 5:**
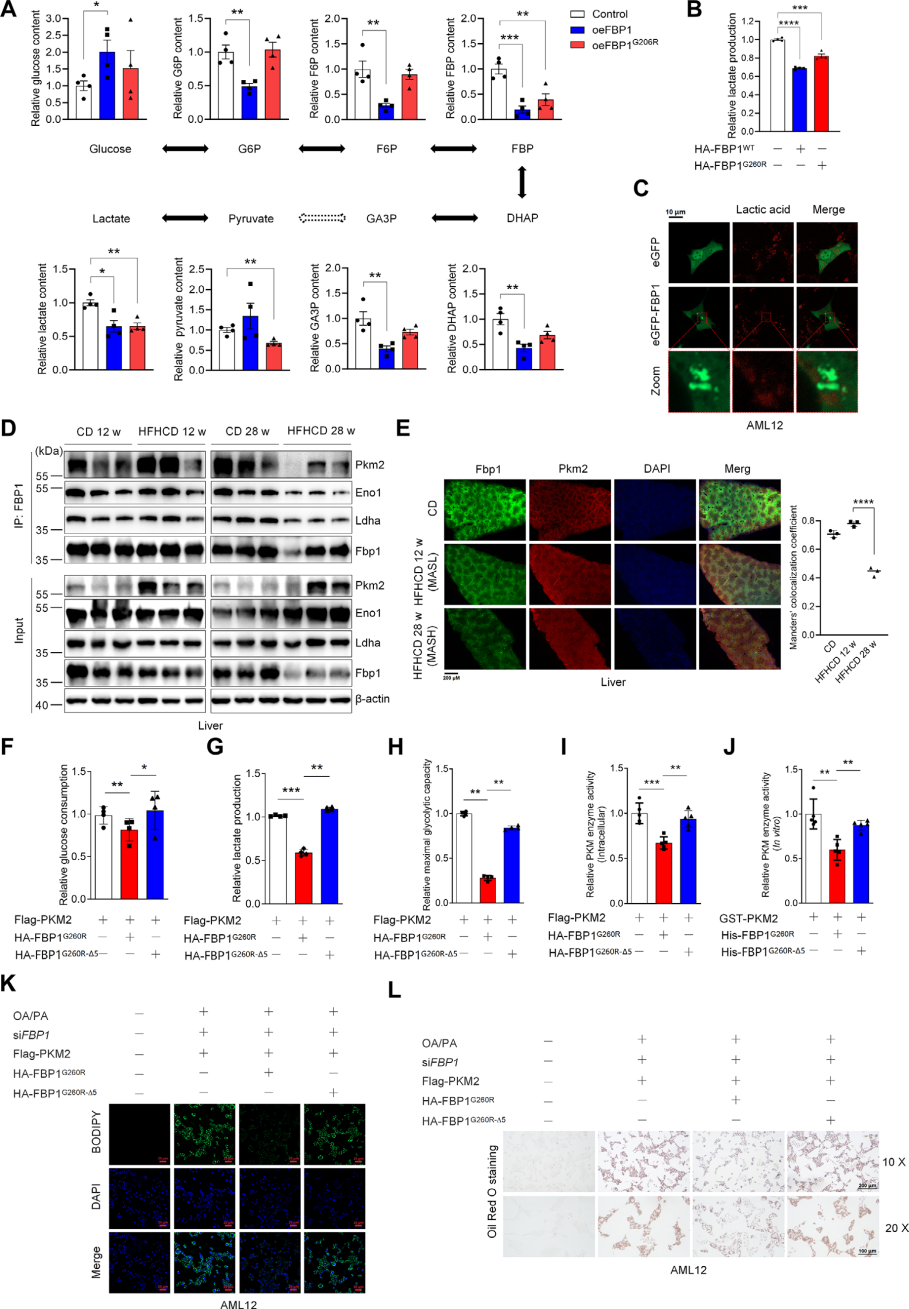
FBP1 Suppresses Hepatic Glycolysis and Lipid Accumulation by Inhibiting PKM2 Activity. (A) Targeted metabolomics quantification of intracellular glycolysis‐related metabolites in AML12 cells overexpressing empty vector (pLVX), wild‐type FBP1, or catalytically inactive FBP1 G260R. Eight key glycolysis‐related metabolites covering upstream, intermediate, and downstream nodes of the full glycolytic pathway were detected, including glucose, glucose‐6‐phosphate (G6P), fructose‐6‐phosphate (F6P), fructose‐1,6‐bisphosphate (FBP), dihydroxyacetone phosphate (DHAP), glyceraldehyde‐3‐phosphate (GA3P), pyruvate, and lactate. (*n* = 4 per group). (B) Lactate production in AML12 cell culture supernatants, measured as the terminal output indicator of the glycolytic pathway (*n* = 4 per group). (C) In situ fluorescence imaging of intracellular lactate in AML12 cells overexpressing eGFP‐FBP1. Green: eGFP‐FBP1; red: lactate fluorescent probe. Scale bars: 10 µm. (D) Endogenous co‐immunoprecipitation showing dynamic interaction between Fbp1 and glycolytic enzymes (Pkm2, Eno1, Ldha) in liver tissues from control diet (CD), 12‐week HFHCD (MASL), and 28‐week HFHCD (MASH) mice. (E) Immunofluorescence co‐localization of Fbp1 (green) and Pkm2 (red) in mouse liver tissues, and quantitative analysis of Manders’ co‐localization coefficient (*n* = 3 per group). Scale bars: 200 µm. (F–H) Glucose consumption (F), lactate production (G), and maximal glycolytic capacity (H) in THLE‐2 cells transfected with Flag‐PKM2 alone, or co‐transfected with HA‐FBP1 G260R or HA‐FBP1 G260R‐Δ5 (*n* = 4 per group). (I) Intracellular PKM activity in THLE‐2 cells with the above treatments (*n* = 4 per group). (J) In vitro recombinant PKM activity assay. Purified GST‐PKM2 was pre‐incubated with recombinant His‐FBP1 G260R or His‐FBP1 G260R‐Δ5 in a standard reaction system containing all essential cofactors and substrates, and enzymatic activity was detected via the coupled enzymatic method (*n* = 5 per group). (K) BODIPY staining of neutral lipids in AML12 cells treated with 0.5 mm OA/PA for 24 h. Scale bars: 20 µm. (L) Oil Red O staining of lipid droplets in AML12 cells treated with 0.5 mm OA/PA for 24 h. Scale bars: 200 µm (10 × magnification), 100 µm (20 × magnification). β‐Actin served as the loading control for all Western blot assays. Nuclei were stained with DAPI for all immunofluorescence assays. Data are presented as mean ± SEM. Significance was assessed by one‐way ANOVA followed by Tukey's test for (A, B, E–J). ^*^ *p* < 0.05, ^**^ *p* < 0.01, ^***^ *p* < 0.001, ^****^ *p* < 0.0001 vs. control group.

To systematically clarify whether the regulatory effect of FBP1 on glycolysis is entirely dependent on its enzymatic catalytic activity, and to dissect the respective contributions of its canonical catalytic function and non‐canonical moonlighting function to glycolytic regulation, we generated the catalytically inactive FBP1 G260R mutant [19] (Figure S5A). Co‐immunoprecipitation and immunofluorescence assays confirmed that the FBP1 G260R mutant neither disrupts its binding capacity to pyruvate kinase M2 (PKM2) nor impairs the formation of compartmentalized complexes (Figure S5B–D). We subsequently performed targeted metabolomics analysis in AML12 hepatocytes overexpressing empty vector, wild‐type FBP1, or catalytically inactive FBP1 G260R, respectively. Quantification of metabolites covering all upstream and downstream nodes of the entire glycolytic pathway revealed that compared with the control group, overexpression of wild‐type FBP1 significantly reduced the intracellular concentrations of 6 core glycolytic metabolites, including glucose‐6‐phosphate (G6P), fructose‐6‐phosphate (F6P), fructose‐1,6‐bisphosphate (FBP), dihydroxyacetone phosphate (DHAP), glyceraldehyde‐3‐phosphate (GA3P) and lactate, while significantly increasing intracellular glucose content, suggesting overall suppression of glycolytic pathway activity (Figure 5A). Notably, compared with the control group, the catalytically inactive FBP1 G260R mutant still significantly downregulated the intracellular levels of most of the aforementioned metabolites, including FBP, DHAP, GA3P, pyruvate, and lactate (Figure 5A). Heatmap analysis of all detected glycolysis‐related metabolites showed that most metabolites across pathway nodes exhibited a consistent gradient change trend (Figure S5E). This result confirms that FBP1 retains glycolytic inhibitory capacity even when its catalytic activity is completely abolished. Furthermore, detection of lactate production in cell culture supernatants was consistent with the metabolomics data: overexpression of wild‐type FBP1 significantly reduced cellular lactate production, while the catalytically inactive FBP1 G260R mutant still markedly inhibited lactate synthesis (Figure 5B). This result further verifies that FBP1 possesses a non‐canonical compartmentalization function independent of catalytic activity, which can independently suppress glycolytic pathway activity and reduce end‐product accumulation.

Collectively, these results indicate that the inhibitory effect of FBP1 on the glycolytic pathway is mediated by two independent regulatory modules: one relies on its classic gluconeogenic catalytic activity, and the other is independent of catalytic function, presumably exerted via FBP1 binding to glycolytic enzymes and subsequent assembly of compartmentalized complexes.

To further clarify the local glycolytic activity and potential metabolite channeling effect within the compartmentalized complexes formed by FBP1 and glycolytic enzymes, we performed in situ fluorescence imaging of intracellular lactate using a specific fluorescent probe. The results showed that in AML12 cells overexpressing eGFP‐FBP1, the lactate fluorescence signal within FBP1‐positive cytoplasmic punctate structures was significantly weaker than that in the surrounding cytoplasm, presenting a clear spatial negative correlation (Figure 5C). This in situ experimental evidence demonstrates that glycolytic enzymes are functionally inhibited after being recruited into FBP1‐mediated complexes, corroborating that these structures act as inhibitory sequestration sites.

To investigate the functional significance of the interaction between Fbp1 and glycolytic enzymes in the progression of MASLD, we first validated their endogenous binding in vivo using liver tissues from HFHCD‐induced mouse models. At the MASL stage (12 weeks of HFHCD feeding), the total protein expression levels of Pkm2, Eno1, and Ldha were either elevated or unchanged; notably, the interactions between endogenous Fbp1 and all three glycolytic enzymes remained stable or slightly enhanced. As the disease progressed to MASH (28 weeks of HFHCD feeding), although the total protein levels of Pkm2, Eno1, and Ldha were all increased in the liver, their co‐immunoprecipitation signals with endogenous Fbp1 were significantly attenuated instead, showing completely inverse trends between total enzyme expression and the detected level of protein association (Figure 5D and Figure S5F–L). Consistent with the co‐IP results, immunofluorescence staining of liver sections showed strong colocalization between endogenous Fbp1 and Pkm2 in the livers of control diet‐fed mice, which was slightly enhanced in the MASL group (HFHCD 12 weeks) but significantly decreased in the MASH group (HFHCD 28 weeks) compared with the MASL group (Figure 5E). This suggests that the cellular abundance of FBP1‐glycolytic enzyme complexes is closely associated with MASLD progression, and the reduction of in vivo complex formation at the MASH stage may further exacerbate progression.

We next investigated whether the inhibitory effect of FBP1 on glycolysis is dependent on its interaction with PKM2. To exclude the effect of FBP1's intrinsic gluconeogenic enzyme activity on cellular glycolytic flux, we constructed an FBP1 G260R enzyme‐inactive mutant and its interaction‐deficient mutant FBP1 G260R‐Δ5 to evaluate the effect of the FBP1‐PKM2 interaction on hepatocyte glycolytic flux. We measured glucose consumption and lactate production in THLE‐2 cells expressing Flag‐PKM2 alone, or co‐expressing HA‐FBP1 enzyme‐inactive mutant (G260R) or its interaction‐deficient mutant G260R‐Δ5 (Figure S5M). Co‐expression of FBP1 G260R, but not G260R‐Δ5, significantly reduced glucose consumption, lactate production, and maximal glycolytic capacity in PKM2‐overexpressing THLE‐2 cells (Figure 5F–H). The Δ5 mutant specifically disrupts FBP1 binding to PKM2 but preserves its interactions with ENO1 and LDHA (Figure S5N), confirming that PKM2 binding is the primary mediator of FBP1's glycolytic repression. These results indicate that FBP1 inhibits glycolysis in a PKM2 interaction‐dependent manner. Intracellular and in vitro PKM activity assays showed that FBP1 G260R, but not G260R‐Δ5, directly inhibited PKM2 enzymatic activity (Figure 5I, J), confirming that FBP1 directly inhibits PKM2 activity through its interaction domain. These results collectively demonstrate that FBP1 reduces PKM2 enzymatic activity through direct interaction, thereby inhibiting glycolytic levels in hepatocytes.

To further distinguish the contributions of direct protein‐protein interaction and spatial compartmentalization, we generated the compartmentalization‐deficient G260R‐Δ7 mutant on the catalytically inactive background. This mutant retains PKM2‐binding capacity but loses the ability to form cytoplasmic compartmentalized complexes (Figure 4B, C). Functionally, G260R‐Δ7 exhibited markedly attenuated glycolytic inhibitory activity, with glucose consumption levels comparable to those of the binding‐deficient G260R‐Δ5 mutant and even higher lactate production (Figure S5O, P). These findings indicate that the assembly of compartmentalized complexes, rather than direct binding alone, mediates the glycolytic suppressive effect, and that protein interaction is critical for recruiting PKM2 into the sequestering structures.

We next investigated the effect of FBP1 on lipid droplet formation in oleic acid/palmitic acid (OA/PA)‐induced steatotic hepatocytes. To exclude the causal role of endogenous FBP1, we performed *Fbp1* knockdown followed by mutant reconstitution assays in AML12 hepatocytes (Figure S5Q). Results from both BODIPY lipid droplet staining and Oil Red O staining demonstrated that, compared with cells reconstituted with the binding‐deficient FBP1 G260R‐Δ5 mutant, reconstitution of the catalytically inactive FBP1 G260R mutant suppressed lipid droplet formation and reduced intracellular lipid accumulation (Figure 5K, L and Figure S5R). These results demonstrate that G260R reconstitution effectively reverses the exacerbated steatosis phenotype, whereas the binding‐deficient G260R‐Δ5 mutant lacks this protective effect. This conclusion was further validated in HepG2 cells (Figure S5S). This indicates that FBP1 inhibits PKM2 activity and glycolysis in a catalytic‐independent manner, thereby suppressing hepatocyte lipid accumulation.

Taken together, these data demonstrate that FBP1 inhibits PKM2 activity through direct interaction, thereby suppressing glycolysis and subsequent lipid accumulation in hepatocytes. The decreased FBP1 expression and weakened interaction with PKM2 during MASLD progression may lead to enhanced glycolysis and hepatic steatosis, revealing a mechanistic link between FBP1 downregulation and metabolic dysregulation in MASLD progression.

### 3.6 Disruption of FBP1‐PKM2 Interaction Exacerbates Diet‐Induced MASL‐to‐MASH Progression In Vivo

**FIGURE 6:**
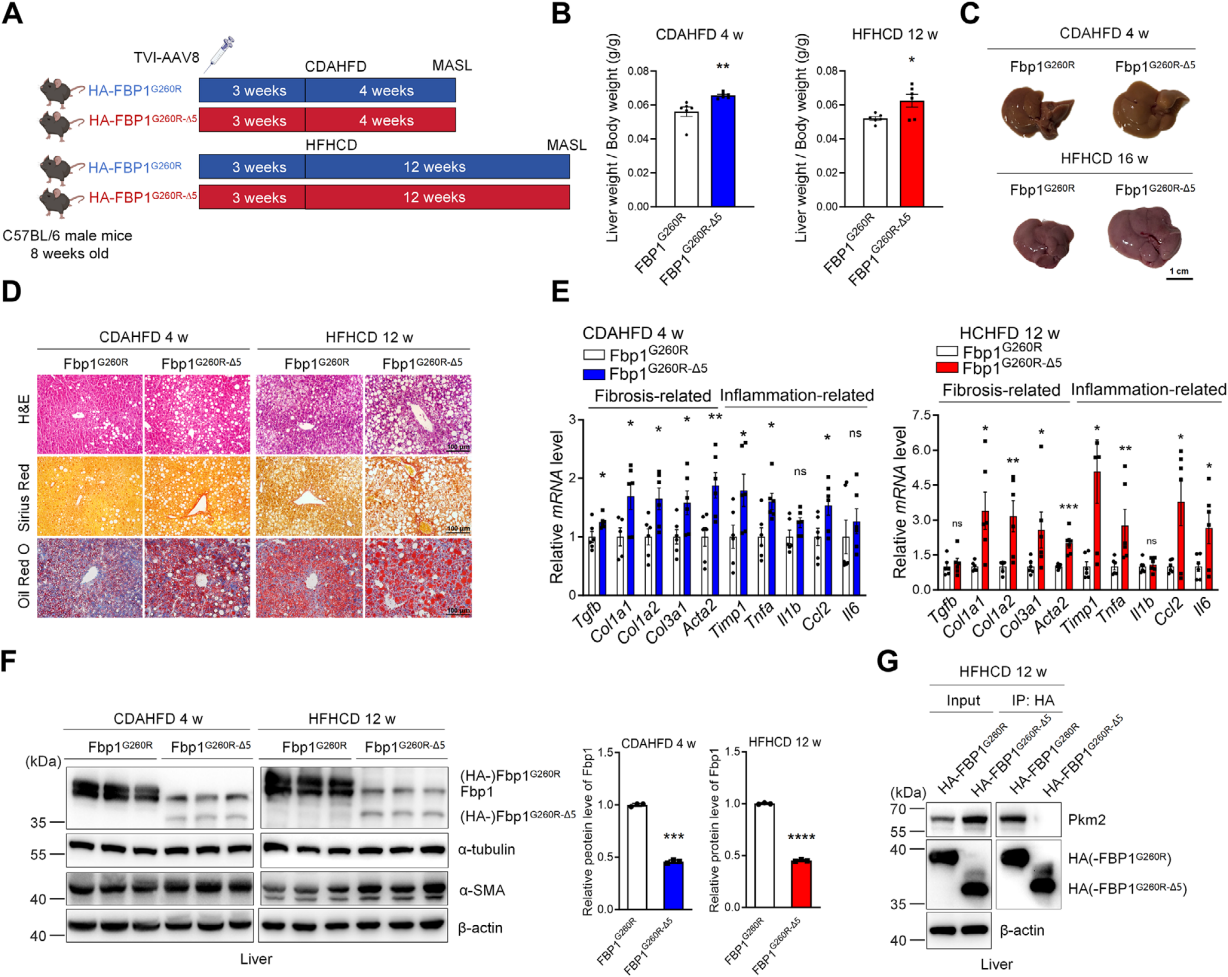
Hepatic Overexpression of PKM2 Interaction‐deficient FBP1 Mutant Exacerbates MASL‐to‐MASH Progression In Vivo. (A) Schematic of in vivo experimental design: 8‐week‐old male C57BL/6 mice were intravenously injected with AAV8‐HA‐FBP1^G260R^ or AAV8‐HA‐FBP1^G260R‐Δ5^, then fed CDAHFD for 4 weeks or HFHCD for 12 weeks after 3 weeks of virus expression (*n* = 6 per group). (B) Liver‐to‐body weight ratios of mice in each group (*n* = 6 per group). (C) Gross morphological appearance of liver tissues. (D) Histopathological analysis: H&E (general histology/steatosis), Sirius Red and Oil Red O staining. Scale bars: 100 µm. (E) qPCR analysis of fibrosis‐ and inflammation‐related gene mRNA levels in liver tissues (*n* = 6 per group). (F) WB analysis of Fbp1 and α‐SMA protein expression in liver tissues, and quantitative analysis of endogenous Fbp1 protein levels in CDAHFD or HFHCD mice (*n* = 3 per group). (G) In vivo co‐IP showing interaction between HA‐FBP1^G260R^/HA‐FBP1^G260R‐Δ5^ and PKM2 in liver tissues. β‐actin and α‐tubulin served as loading controls. Data are presented as mean ± SEM. Significance was assessed by a two‐sided unpaired Student's *t*‐test for (B, E, F). ^*^ *p* < 0.05, ^**^ *p* < 0.01, ^***^ *p* < 0.001, ^****^ *p* < 0.0001 vs. control group.

To validate the in vivo function of the FBP1‐PKM2 interaction, we delivered AAV8 encoding HA‐FBP1 G260R or G260R‐Δ5 to mouse livers, followed by CDAHFD (4 weeks) or HFHCD (12 weeks) feeding to monitor hepatic steatosis and inflammation in diet‐induced MASL‐to‐MASH models (Figure 6A and Figure S6A, B). In both models, FBP1 G260R‐Δ5 overexpression resulted in higher liver‐to‐body weight ratios, enlarged pale livers, increased hepatocyte ballooning, inflammation, collagen deposition, and lipid accumulation compared with FBP1 G260R (Figure 6B–D).

qPCR analysis of liver tissues further confirmed that, in both CDAHFD and HFHCD induced MASL‐to‐MASH models, overexpression of FBP1 G260R‐Δ5 led to significant upregulation of liver fibrosis‐related genes such as *Tgfb*, *Col1a1*, *Col1a2, Col3a1*, *Acta2*, and *Timp1*, as well as inflammation‐related genes such as *Tnfa*, *Il1b*, *Ccl2*, and *Il6* in mouse livers compared with the FBP1 G260R overexpression group (Figure 6E). Western blot analysis also showed that the expression level of α‐SMA, a key marker of hepatic stellate cell activation and fibrosis, was increased in the livers of the FBP1 G260R‐Δ5 group (Figure 6F and Figure S6C). Meanwhile, compared with the FBP1 G260R overexpression group, FBP1 G260R‐Δ5 overexpression resulted in a significant decrease in endogenous Fbp1 protein expression in mouse livers (Figure 6F), consistent with our previous observation that FBP1 expression levels decrease with progression (Figure 1). Notably, liver tissue co‐IP experiments confirmed that only FBP1 G260R interacted with PKM2, while FBP1 G260R‐Δ5 completely lost this binding ability, further demonstrating that blocking the FBP1‐PKM2 interaction promotes MASLD progression (Figure 6G). To rule out the interference of FBP1 gluconeogenic catalytic activity on phenotypic differences, we further measured gluconeogenesis‐related metabolic parameters. Mice in the G260R‐Δ5 group exhibited significantly decreased hepatic glycogen content, as well as elevated fasting blood glucose and serum insulin levels (Figure S6D–F). These findings indicate that the above metabolic abnormalities are secondary changes triggered by more severe MASLD and insulin resistance, rather than primary alterations directly caused by differences in FBP1 catalytic activity.

In summary, these in vivo results demonstrate that the protective effect of FBP1 against diet‐induced hepatic steatosis, inflammation, and fibrosis depends on its interaction with PKM2. The FBP1 G260R‐Δ5 mutant disrupts this interaction, significantly exacerbating the severity of hepatic steatosis, inflammation, and fibrosis, leading to further deterioration of MASLD progression.

## 4 Discussion

**FIGURE 7:**
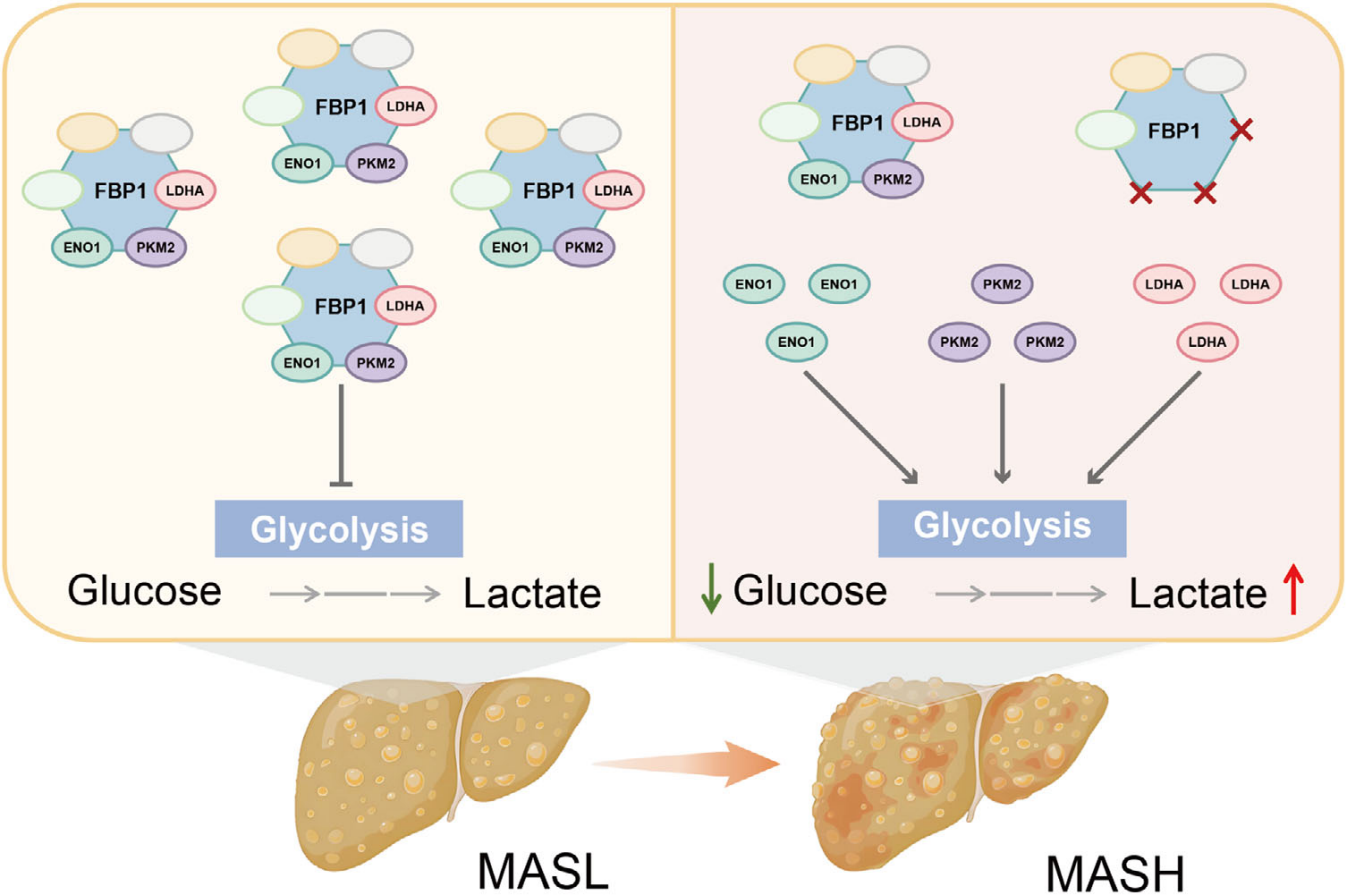
FBP1‐mediated glycolytic enzyme compartmentalization regulates hepatocyte glycolysis during MASLD progression. In normal hepatocytes, FBP1 forms compartmentalized complexes with PKM2, ENO1, and LDHA independent of its catalytic activity, inhibiting PKM2 activity and restricting excessive glycolysis. During MASLD progression, FBP1 downregulation markedly reduces the abundance of these complexes and releases the “hijacked” glycolytic enzymes, leading to PKM2 activation, enhanced glycolytic reprogramming, and subsequent hepatic steatosis, inflammation, and fibrosis. Thus, targeting the FBP1‐PKM2 interaction may represent a promising candidate intervention strategy for MASLD.

Hepatic glycolytic reprogramming contributes to MASLD progression. This metabolic reprogramming not only provides substrates for *de novo* lipogenesis but also promotes inflammation and fibrosis progression, further accelerating the MASL‐to‐MASH transition in the liver [45]. Despite extensive research on MASLD, the molecular mechanisms underlying metabolic reprogramming characterized by excessive glycolysis during MASLD progression remain incompletely elucidated, and no clinically validated therapeutic targets are currently available. This study identifies a novel non‐enzymatic function of the gluconeogenic enzyme FBP1: it directly interacts with PKM2, ENO1, and LDHA to form dynamic compartmentalized complexes, suppressing PKM2 activity and excessive glycolysis. Progressive FBP1 downregulation during the MASL‐to‐MASH transition markedly reduces the abundance of these complexes and releases the FBP1‐“hijacked” glycolytic enzymes, leading to enhanced glycolysis and accelerated disease progression (Figure 7).

FBP1 expression is universally silenced in both human and mouse liver tumors, while the dynamic expression pattern of FBP1 during the progression from MASL to MASH remains unclear. In this study, we observed progressive downregulation of FBP1 in both the CDAHFD and HFHCD preclinical MASLD models, as well as in clinical specimens. Mechanistically, hepatocyte nuclear factor 4α (HNF4α), as the core transcription factor that directly binds and activates the *FBP1* promoter to maintain hepatic glucose homeostasis, undergoes nuclear export under MASLD‐related microenvironmental stresses, including lipotoxicity, chronic inflammation, and oxidative stress; our results further confirm that impaired HNF4α nuclear localization is a key upstream driver of FBP1 transcriptional downregulation. Besides HNF4α, other microenvironment‐responsive transcription factors such as HIF‐1α may also participate in FBP1 regulation: FBP1 has been confirmed to inhibit HIF‐1α transcriptional activity, and the two likely form a pathological positive feedback loop in the hypoxic and acidified MASLD liver microenvironment to amplify disease progression. Multiple additional regulatory mechanisms may also be involved, including transcriptional silencing induced by promoter hypermethylation, post‐translational degradation mediated by oxidative stress and inflammatory pathways, and loss of p53‐mediated transcriptional activation, all of which warrant further in‐depth investigation [16]. Notably, FBP1 downregulation and MASLD progression do not follow a simple unidirectional cause‐effect relationship, but form a pathological positive feedback loop: MASLD pathological microenvironments downregulate FBP1 expression via impairing HNF4α nuclear localization, while FBP1 reduction in turn relieves glycolytic inhibition and exacerbates hepatocyte steatosis, inflammation, and fibrosis, further accelerating disease deterioration. This vicious cycle jointly drives the transition from MASL to MASH. An in‐depth understanding of these mechanisms provides an important theoretical basis for the diagnosis and targeted intervention of the MASL‐to‐MASH transition and its associated liver cancer.

Traditionally, FBP1 is known for its gluconeogenic catalytic function. In this study, we demonstrate that FBP1 also acts as a key negative regulator of glycolysis via non‐enzymatic protein‐protein interactions. This cross‐regulation between gluconeogenic and glycolytic enzymes represents a novel mechanism for maintaining hepatic metabolic homeostasis. Interestingly, this study found that FBP1 drives the assembly of dynamic glycolytic compartmentalized complexes, a novel regulatory mode of hepatic glycolysis. The 276–338 aa domain of FBP1 is critical for complex formation, which may involve weak hydrophobic interactions. These complexes are present in both normal and cancer cells, suggesting a universal role in metabolic regulation.

Strikingly, FBP1‐mediated compartmentalized complexes are functionally distinct from classic metabolism‐promoting glycolytic aggregates. FBP1 acts as an active driver of complex formation rather than a passive component incorporated into pre‐existing aggregates, and sequestration of glycolytic enzymes within these complexes consequently suppresses glycolytic flux. Our experimental data confirm that these structures are not non‐specific protein aggregates: FBP1 forms dynamic, hexanediol‐sensitive punctate assemblies in cells, and FBP1 effectively inhibits the catalytic activity of sequestered glycolytic enzymes. Structural docking analysis further verifies that FBP1 binds near the active site and tetramerization interface of PKM2, exerting this inhibitory effect through steric hindrance. Therefore, FBP1‐mediated compartmentalized complexes are essentially inhibitory sequestration sites, rather than active functional compartments that drive glycolytic metabolism.

Consistent with our KEGG enrichment results, among the enriched functional categories, carbon metabolism and biosynthesis of amino acids largely share the same set of glycolytic/gluconeogenic enzymes rather than representing independent functional clusters, while tight junction‐annotated proteins are mainly cytoskeletal regulators and membrane trafficking factors, suggesting an indirect functional association with FBP1. Our study prioritizes glycolytic compartmentalization because it shows the highest enrichment significance, is intrinsically linked to FBP1's canonical metabolic function, and corresponds to the core glycolytic reprogramming hallmark of MASLD progression, and this regulatory axis has also been systematically validated by multiple orthogonal assays. Further dissection of the glycolysis pathway reveals that FBP1 preferentially assembles proximal complexes with downstream glycolytic enzymes, while HK2 and key PPP enzymes are not enriched in its proximity interactome. This selective compartmentalization may alter flux partitioning at glucose metabolic branch points; while our targeted metabolomics data show reduced levels of most glycolytic intermediates, consistent with overall glycolytic suppression and no net accumulation of upstream metabolites, we cannot exclude altered flux distribution into auxiliary pathways such as the PPP. The remaining enriched pathways and potential PPP flux redistribution serve as valuable hypothesis‐generating clues for FBP1's broader hepatic metabolic roles and await systematic verification in future studies.

Furthermore, protein interactions, post‐translational modifications, and cellular microenvironment signals collectively participate in the dynamic regulation of glycolytic enzyme compartmentalized complexes [33, 46, 47, 48]. Further exploration of the dynamic regulatory mechanisms of FBP1‐mediated glycolytic enzyme compartmentalization and resolution of the complete composition of these compartmentalized complexes hold profound scientific significance and translational clinical value. This finding provides a new molecular target and theoretical basis for targeted intervention of metabolic‐related diseases such as MASLD and tumors.

PKM2 exists in a dynamic equilibrium between enzymatically active tetramers and inactive dimers/monomers, and this equilibrium is tightly controlled by interactions with the C‐terminal domain [49, 50, 51]. FBP1 binds to the C‐terminal catalytic domain (amino acids 390–531) of PKM2, which harbors both the active site and the tetramerization interface. We have further clarified the relative contributions of distinct regulatory modes, and FBP1 may regulate PKM2 function through three complementary layers. First, the direct FBP1‐PKM2 association intrinsically inhibits PKM2 enzymatic activity; however, this effect alone is insufficient to exert robust glycolytic suppression. Second, and more prominently, FBP1 sequesters PKM2 within cytoplasmic compartmentalized complexes. FBP1‐mediated compartmentalization not only restricts PKM2 participation in the glycolytic pathway, but may also concentrate and stabilize this inhibitory protein‐protein interaction, which accounts for the bulk of the glycolytic suppressive effect. Direct protein‐protein interaction primarily serves as the molecular basis for recruiting PKM2 into these complexes. Third, results from nuclear‐cytoplasmic fractionation assays further confirm that FBP1‐mediated compartmentalization impedes the nuclear translocation of PKM2, which may in turn attenuate its function as a nuclear transcriptional co‐activator under pathological conditions. Nevertheless, in the pathological context of MASLD, the pathophysiological roles of specific downstream transcriptional programs regulated by nuclear‐localized PKM2 remain to be further validated by in vivo experiments.

Consistent with this regulatory model, we further uncovered a dynamic pattern of the interaction between FBP1 and glycolytic enzymes during the progression of MASLD. At the early MASL stage, the formation of FBP1‐glycolytic enzyme complexes is compensatorily enhanced as a hepatic protective mechanism to restrain excessive glycolytic activation; conversely, the cellular level of these protein complexes decreases significantly at the late MASH stage. Loss of FBP1‐mediated compartmentalized inhibition, coupled with the global upregulation of glycolytic enzyme expression, ultimately leads to aberrant hyperactivation of glycolytic metabolic reprogramming and exacerbates disease progression. This dynamic change in protein complex abundance may arise from multiple factors, including reduced FBP1 expression and altered microenvironmental metabolites, which in turn modulate the assembly of the protein complexes. Dissecting the precise molecular mechanisms governing their binding affinity holds promise for delivering novel actionable targets and intervention strategies for the precise treatment of MASLD.

Some limitations of the current study should be acknowledged. The fine mechanistic dissection of these compartmentalized complexes relies on an overexpression system widely used in the field of biomolecular compartmentalization research; the dynamic properties of endogenous low‐abundance complexes still require further verification via super‐resolution imaging techniques and precise point mutation knock‐in animal or cell models in the future. In addition, the complete molecular composition and full regulatory network of these compartmentalized complexes have not been fully elucidated.

Regarding the clinical cohort, the current sample size is relatively limited, and confounding factors such as enrichment of type 2 diabetes in the MASH group may interfere with the correlation analysis. Only univariate correlation analysis has been performed to date, and the independence of the association between FBP1 and disease severity still needs to be verified by confounder‐adjusted multivariable regression in a larger independent cohort.

Notably, all in vivo validation experiments in this study adopted a preventive intervention mode synchronized with disease modeling, which only confirmed that FBP1 delays the onset and progression of MASLD and exerts a hepatoprotective effect. Whether restoring the binding function between FBP1 and glycolytic enzymes can reverse established steatosis, inflammation, and fibrosis in MASH remains to be verified by therapeutic reversal experiments in follow‐up studies.

Follow‐up studies will systematically characterize the full component spectrum and spatiotemporal regulatory mechanisms of the complexes, and expand the clinical sample size to further clarify the pathophysiological role and clinical translational value of FBP1‐mediated compartmentalization regulation in MASLD progression.

In conclusion, our study establishes a complete pathophysiological evidence chain from molecular interaction, metabolic reprogramming, cellular and in vivo phenotypes to clinical correlation, and identifies the FBP1‐PKM2 axis as a promising candidate intervention target for MASLD. Enhancing FBP1‐PKM2 interaction stability or promoting the assembly of FBP1‐mediated glycolytic inhibitory complexes may represent effective intervention strategies for MASLD. This work also highlights the translational value of targeting the non‐enzymatic spatial regulatory functions of metabolic enzymes, expanding the understanding of moonlighting functions of metabolic enzymes in chronic liver diseases.

## Author Contributions

**Busong Wang**, **Yiping Tang**, **Chaojun Li**, **Lingdong Kong** and **Lei Fang**: conceptualization. **Busong Wang**: methodology. **Yiping Tang** and **Yuxin Chen**: formal analysis. **Busong Wang**, **Yiping Tang**, **Yuxin Chen**, and **Na Li**: investigation. **Xuening Dai**, **Jing Wu**, and **Bin Xue**: resources. **Busong Wang**, **Xuening Dai**, **Jing Wu**, **Bin Xue**, and **Yuxin Chen**: data curation. **Busong Wang**: writing – original draft. **Jingzi Zhang** and **Lei Fang**: writing – review and editing. **Yiping Tang** and **Na Li**: visualization. **Jingzi Zhang**, **Chaojun Li**, **Lingdong Kong**, and **Lei Fang**: supervision. **Chaojun Li** and **Lei Fang**: project administration. **Jingzi Zhang**, **Lingdong Kong**, and **Lei Fang**: funding acquisition.

## Funding

This work was supported by grants from the National Natural Science Foundation of China (Grant Nos. 32071256 and 32371336 to Lei Fang), the Youth Fund of the National Natural Science Foundation of China (Grant No. 32201066 to Jingzi Zhang), the Natural Science Foundation of Jiangsu Province (Grant No. BK20221443 to Lei Fang), and the Major Science and Technology Special Project of Jiangsu Province (Grant No. BG2025036).

## Ethics Approvals

The animal experiments were approved by the Animal Ethics Committee of Nanjing University (Approval No. IACUC‐D2303045) and conducted in compliance with all relevant ethical regulations regarding animal research. All human tissue samples were obtained with approval from the Medical Ethics Subcommittee of the Nanjing University Science and Technology Ethics Committee (Approval No. AP20230310008). Written informed consent was obtained from all participants prior to the start of the study. This study was a retrospective observational analysis of de‐identified clinical biopsy specimens.

## Conflicts of Interest

The authors declare no conflicts of interest.

## Supporting information




**Supporting File**: advs77875‐sup‐0001‐SuppMat.docx.

## Data Availability

The mass spectrometry proteomics data have been deposited in the ProteomeXchange Consortium (https://proteomecentral.proteomexchange.org) via the iProX partner repository with the dataset identifier PXD079036.
