# Supplementary material for: FBP1‐Mediated Compartmentalization of Glycolytic Enzyme Complexes Suppresses PKM2 Function to Ameliorate MASLD Progression
Source: Adv Sci (Weinh). 2026 Sep 27:e77875. Online ahead of print. doi: 10.1002/advs.77875 (PMC13616314; doi:10.1002/advs.77875)
Supplement: Supplementary file 1 [file ADVS-9999-e77875-s001.docx]

**FBP1-mediated Compartmentalization of Glycolytic Enzyme Complexes Suppresses PKM2 Function to Ameliorate MASLD Progression**

Busong Wang, Yiping Tang, Na Li, Xuening Dai, Jing Wu, Bin Xue, Yuxin Chen, Jingzi Zhang, Lingdong Kong, Chaojun Li, Lei Fang

**Table of contents**

**Supplementary materials and methods ........................................................... 2**

**Supplementary figures .................................................................................... 18**

**Supplementary tables ...................................................................................... 25**

**Supplementary references .............................................................................. 29**

**Supplementary materials and methods**

## 1. Study Population and Ethical Approval

This retrospective case-control study was approved by the Medical Ethics Subcommittee of the Nanjing University Science and Technology Ethics Committee (Approval No. AP20230310008). Written informed consent was obtained from all enrolled participants. Patients diagnosed with **MASLD** who underwent liver biopsy at the Department of Bariatric Surgery, the First Affiliated Hospital of Nanjing Medical University between January 2024 and December 2025 were included.

## 2. Study Design and 1:1 Propensity Score Matching

A 1:1 propensity score matching (PSM) was performed to balance confounding factors. Matching variables included: sex (exact match) and age (± 5 years). In total, 20 patients were matched into 10 pairs, comprising 10 patients with **MASL** and 10 with **MASH**.

## 3. Histological Evaluation and Patient Grouping

Liver biopsy specimens were fixed in 10% neutral buffered formalin, paraffin-embedded, serially sectioned, and stained with hematoxylin-eosin (HE), Masson trichrome, and reticulin stains. All specimens were independently evaluated in a blinded manner by two liver pathologists with > 10 years of experience; discrepancies were resolved by consensus review.

Histological scoring was performed using validated systems: **NAFLD Activity Score (NAS, NASH-CRN)**^2^: Steatosis (S: 0-3), lobular inflammation (L: 0-3), hepatocellular ballooning (B: 0-2); total NAS = S+L+B (0-8). **SAF scoring system (EASL-recommended)**^3^: For borderline NAS 3-4 cases; activity score (A) = L+B.

**Fibrosis staging**: METAVIR criteria (F0-F4). Final grouping: MASL group: NAS < 3, or NAS 3-4 with SAF activity score A=0; MASH group: NAS ≥ 5, or NAS 3-4 with S ≥ 1 and A ≥ 1. Detailed clinical and histological data are listed in Supplementary Table S1.

## 4. Animal Experiments and Ethical Approval

Male C57BL/6J mice (8 weeks old) were purchased from GemPharmatech Co., Ltd. (Nanjing, China; Cat. No. N000013). Mice were housed in a specific pathogen-free (SPF) facility under a 12 h light/dark cycle, 22±2 °C, 50±10% humidity, with ad libitum access to sterilized chow and water. All animal procedures were conducted in accordance with the NIH Guide for the Care and Use of Laboratory Animals and the ARRIVE 2.0 guidelines. Mice were euthanized by isoflurane overdose followed by cervical dislocation.

All animal experimental procedures were reviewed and approved by the Animal Ethics Committee of Nanjing University (Approval No. IACUC-D2303045) and were performed in compliance with all relevant ethical regulations regarding animal research.

## 5. Mouse Models of MASLD

Before model establishment, mice were randomly assigned to control or different diet groups based on body weight to minimize baseline phenotypic bias. Cage positions were randomly distributed across the SPF facility and rotated weekly to eliminate potential cage effect.

Three diet-induced MASLD models were established: **CDAHFD model**: Mice were fed a choline-deficient, amino-acid-defined, high-fat diet (Jiangsu Synergy Bioengineering Co., Ltd., Yangzhou, China; Cat. No. XTMRCD60) for 6 weeks (MASL: 4 weeks; MASH: 6 weeks). **HFHCD model**: Mice were fed a high-fat, high-cholesterol diet (Jiangsu Synergy Bioengineering Co., Ltd.; Cat. No. XT310) for 28 weeks (MASL: 12 weeks; MASH: 28 weeks). **WD model**: Mice were fed a Western diet (Jiangsu Synergy Bioengineering Co., Ltd.; Cat. No. XT108C) for 24 weeks (MASL: 14 weeks; MASH: 24 weeks).

Age-matched control mice were fed standard chow (Jiangsu Synergy Bioengineering Co., Ltd.; Cat. No. XT93G).

Preset exclusion criteria for final analysis were as follows: mice that died unexpectedly during feeding, mice with severe infection or abdominal abscess at sacrifice, and mice with spontaneous liver lesions detected by pathological examination were excluded from downstream statistical analysis.

At sacrifice, mice were fasted for 12 h, anesthetized, and liver tissues were harvested for fixation or snap-freezing in liquid nitrogen. Blood samples were collected, and serum was isolated by centrifugation and stored at -80 °C for subsequent biochemical detection. All histological assessments (including NAS scoring, fibrosis grading and steatosis evaluation) were performed in a single-blinded manner by two independent pathologists who were unaware of group allocation to avoid subjective evaluation bias.

## 6. AAV-Mediated *In Vivo* Gene Delivery

Recombinant adeno-associated virus serotype 8 (AAV8) vectors expressing HA-tagged human FBP1 G260R or FBP1 G260R-Δ5 were constructed and packaged by GeneChem Co., Ltd. (Shanghai, China). Mice received 5×10^11^ viral genomes (vg) *via* tail vein injection at 8 weeks of age. After 3 weeks of transgene expression, mice were subjected to CDAHFD (4 weeks) or HFHCD (12 weeks) feeding.

## 7. Cell Culture and Treatments

Human hepatocyte lines THLE-2, HepG2, HEK293, HEK293T, and mouse AML12 cells were obtained from the American Type Culture Collection (ATCC, Manassas, VA, USA). THLE-2 cells were cultured in Bronchial Epithelial Cell Growth Medium (Procell, Wuhan, China; Cat. No. CM-0833) supplemented with 10% fetal bovine serum (FBS; ExCell Bio, Shanghai, China; Cat. No. FCS500) and 1% penicillin-streptomycin (Gibco, Grand Island, NY, USA; Cat. No. 15140122). HepG2, HEK293, HEK293T, and AML12 cells were cultured in DMEM (Wisent, Saint-Jean-Baptiste, Canada; Cat. No. 319-050-CL) with 10% FBS and 1% penicillin-streptomycin. All cells were incubated at 37 °C with 5% CO₂.

For lipid overload, cells were treated with 0.5 mM oleic acid/palmitic acid (OA/PA, 2:1; Sigma-Aldrich, St. Louis, MO, USA; Cat. Nos. O1383, P5585) for 24 h. For weak interaction inhibition, cells were treated with 1.5% 1,6-hexanediol (Sigma-Aldrich; Cat. No. 240117) for designated durations.

## 8. Plasmid Construction and Mutagenesis

Full-length human FBP1, PKM2, ENO1, and LDHA cDNAs were amplified from a human liver cDNA library. Truncation and point mutants were generated by PCR-based site-directed mutagenesis. Specifically, all domain truncation mutants used in functional assays (including G260R-Δ5 and G260R-Δ7) were generated on the background of the catalytically inactive FBP1 G260R mutant, to exclude any interference from FBP1’s intrinsic gluconeogenic activity. All constructs were verified by Sanger sequencing. For mammalian expression, cDNAs were subcloned into pcDNA3.1-HA, pcDNA3.1-Flag, pEGFP-C1, or pmCherry-C1 vectors. For prokaryotic expression, cDNAs were inserted into pGEX-4T-1 (GST-tag) or pET-28a (His-tag). For proximity labeling, FBP1 was subcloned into pcDNA3.1-HA-TurboID.

**9. Cell Transfection**

**9.1 Plasmid Transfection**

Plasmid transfection was performed using Lipomaster 2000 (Vazyme; Cat. No. TL201-01) according to the manufacturer’s protocol. Briefly, cells were seeded to reach 70%–80% confluence on the day of transfection. Plasmids and transfection reagent were diluted separately in serum-free Opti-MEM medium, mixed gently and incubated for 5 min at room temperature, then added dropwise to the cell culture medium. Equal total plasmid mass was maintained across all groups by supplementing with empty vector to ensure consistent transfection efficiency. Cells were harvested for subsequent experiments 48 h after transfection.

**9.2 siRNA Transfection**

Small interfering RNAs (siRNAs) targeting the 3' untranslated region (3'UTR) of mouse *Fbp1* and negative control siRNA were synthesized by Sangon Biotech Co., Ltd. (Shanghai, China). The sequences were as follows: si-*Fbp1* sense: 5'-GGAUGAGAAUGUGAAGAAUTT-3'; si-*Fbp1* antisense: 5'-AUUCUUCACAUUCUCAUCCTT-3'. siRNA transfection was performed with Lipomaster 2000 at a final siRNA concentration of 50 nM. Cells were collected 48 h after transfection, and knockdown efficiency was verified by Western blot before follow-up experiments.

## 10. Western Blot Analysis

Total protein was extracted using RIPA lysis buffer (Beyotime, Shanghai, China; Cat. No. P0013B) with protease/phosphatase inhibitor cocktails (MCE, Monmouth Junction, NJ, USA; Cat. Nos. HY-K0011, HY-K0021). Protein concentration was quantified by BCA assay (Thermo Fisher Scientific, Waltham, MA, USA; Cat. No. 23227). Equal protein (10 μg) was separated by SDS-PAGE and transferred to PVDF membranes (Vazyme, Nanjing, China; Cat. No. E801-01). Membranes were blocked with 5% non-fat milk in TBST, incubated with primary antibodies overnight at 4 °C, then HRP-conjugated secondary antibodies for 1 h at room temperature. Signals were detected with ECL reagent (Vazyme; Cat. No. E422-01) and visualized using a Tanon 5200 chemiluminescence imaging system.

Antibody information is provided in Supplementary Table S2.

## 11. Nuclear-Cytoplasmic Fractionation

Nuclear and cytoplasmic protein fractions were isolated using a Fresh/Frozen Tissue Cytoplasmic and Nuclear Rapid Isolation Kit (Solarbio, Cat. No. EX3271) following the manufacturer’s protocol with minor adjustments adapted for cell samples. Briefly, transfected AML12 cells were harvested, washed twice with ice-cold PBS, and resuspended in pre-chilled Buffer A supplemented with a protease inhibitor cocktail. After 5 min of incubation on ice, samples were centrifuged at 14,000 × g for 5 min at 4 °C, and the supernatant was collected as the cytoplasmic fraction. The nuclear pellet was washed once with Buffer A, then lysed with Buffer D for denatured nuclear protein extraction. After complete lysis, samples were centrifuged at 14,000 × g for 5 min at 4 °C, and the supernatant was retained as the nuclear fraction. The purity of subcellular fractionation was validated by Western blotting with GAPDH as the cytoplasmic marker and Lamin B1 as the nuclear marker.

## 12. Immunoprecipitation (IP)

For exogenous IP, HEK293T cells were transfected with indicated plasmids using Lipomaster 2000 (Vazyme; Cat. No. TL201-01). After 48 h, cells were lysed in IP buffer (20 mM Tris-HCl pH 7.5, 150 mM NaCl, 1% Triton X-100, 1 mM EDTA) with protease inhibitors. Lysates were incubated with anti-HA antibody overnight at 4 °C, then Protein A/G magnetic beads (Vazyme; Cat. No. VA201-01) for 1 h at room temperature.

For endogenous IP, THLE-2 cells or liver tissues were lysed in IP buffer. Lysates were pre-cleared, then incubated with anti-FBP1 antibody or normal rabbit IgG (negative control) overnight at 4 °C, followed by Protein A/G beads for 4 h. Beads were washed five times, and bound proteins were eluted with 2×SDS loading buffer for Western blot analysis.

## 13. GST Pull-Down Assay

GST- and His-tagged recombinant proteins were expressed in E. coli BL21(DE3) (Vazyme; Cat. No. C504-02) and purified with GST resin (TransGen, Beijing, China; Cat. No. DP201-01) or Ni-NTA agarose (TransGen; Cat. No. DP101-01). GST fusion proteins were eluted with reduced glutathione (MCE; Cat. No. HY-D0187); His fusion proteins with imidazole (MCE; Cat. No. HY-D0837). For pull-down, 5 μg GST/GST-fusion proteins were incubated with 5 μg His-fusion proteins in binding buffer for 2 h at 4 °C, then incubated with GST resin for 1 h. Beads were washed, eluted, and analyzed by Western blot.

## 14. TurboID Proximity Labeling and LC-MS/MS

THLE-2 cells were infected with lentiviruses carrying either HA-TurboID or HA-TurboID-FBP1. At 48 h post-infection, cells were treated with 50 μM biotin (MCE, Cat. No. HY-B0511) for 30 min at 37 °C. Biotinylated proteins were purified using NeutrAvidin agarose resin (Thermo Fisher Scientific; Cat. No. 29202). The purified proteins were separated by SDS-PAGE and stained with Coomassie Brilliant Blue. Gel bands were excised and sequentially subjected to decolorization, reduction, and alkylation, followed by overnight tryptic digestion (Promega, Madison, WI, USA, Cat. no. V528A) at 37 °C. The resulting peptides were extracted, concentrated, and reconstituted for LC-MS/MS analysis using an ACQUITY UPLC M-Class system coupled to a ZenoTOF 7600 mass spectrometer (SCIEX, Framingham, MA, USA). Raw mass spectrometry data were processed with ProteinPilot and ProteinProspector software, and database searches were performed against the SwissProt human database for bioinformatics analysis. Raw mass spectrometry data were processed with ProteinPilot and ProteinProspector, searched against the SwissProt human database. Proteins were identified with a peptide-level FDR < 1% and at least two unique peptides. Differentially enriched proteins were defined by fold change ≥ 1.5, *p* < 0.05. GO and KEGG enrichment analyses were performed to annotate the biological functions of FBP1-interacting proteins.

## 15. Immunofluorescence and Confocal Microscopy

### 15.1 Cell Immunofluorescence

HEK293, AML12 or HepG2 cells on coverslips were fixed with 4% paraformaldehyde (PFA), stained with DAPI (MCE; Cat. No. HY-D0814), and imaged using a Zeiss LSM 900 confocal microscope.

### 15.2 Tissue Immunofluorescence

Liver tissues were fixed in 4% PFA, paraffin-embedded, sectioned (4 μm), and subjected to antigen retrieval. Sections were blocked with 5% BSA, incubated with primary antibodies overnight at 4 °C, then Alexa Fluor 488/594 secondary antibodies (Invitrogen, Waltham, MA, USA; Cat. Nos. A-11031, A-11012). Nuclei were stained with DAPI. Colocalization was quantified with ImageJ using Mander's coefficient.

## 16. Fluorescence Recovery After Photobleaching (FRAP)

HEK293T cells were transfected with eGFP-FBP1 and seeded in glass-bottom dishes. FRAP was performed on a Zeiss LSM 900 microscope (37 °C, 5% CO₂). A region of interest (ROI) containing eGFP-FBP1 puncta was bleached at 100% laser power for 1 s. Fluorescence recovery was recorded every 2 s for 40 s. Intensity was normalized to pre-bleach levels, and curves were plotted with GraphPad Prism 9.

## 17. Histological Staining and Quantification

Liver sections were stained with H&E, Sirius Red, or Oil Red O using commercial kits (H&E: Proteintech, Cat. No. PK10031; Sirius Red & Oil Red O: Solarbio, Beijing, China, Cat. Nos. G1470, G1260). Stained tissue sections were imaged with an Olympus BX53 microscope. BODIPY 493/503 (MCE, Cat. No. HY-W090090) was used for lipid droplet staining in cell samples.

For immunohistochemical staining of FBP1, semi-quantitative evaluation was performed using the immunoreactive score (IRS) system for both mouse and human liver tissues. The IRS was calculated as the product of staining intensity (0 = negative, 1 = weak, 2 = moderate, 3 = strong) and the percentage of positively stained hepatocytes (0 = 0%, 1 = 1–25%, 2 = 26–50%, 3 = 51–75%, 4 = 76–100%), with a total score ranging from 0 to 12. For mouse liver tissues, six random fields were analyzed per mouse with 3 biological replicates per group, and the average IRS per mouse was used for statistical comparison. For the 20 enrolled human liver biopsy specimens (10 MASL and 10 MASH), FBP1 IHC staining was evaluated independently by two board-certified liver pathologists in a blinded manner, and discrepancies in scoring were resolved by consensus review.

## 18. RNA Isolation and Quantitative Real-Time PCR (qPCR)

Total RNA was extracted with RNA isolater (Vazyme; Cat. No. R401-01) and reverse-transcribed into cDNA using HiScript II Q RT SuperMix (Vazyme; Cat. No. R223-01). qPCR was performed with ChamQ Universal SYBR Master Mix (Vazyme; Cat. No. Q711-02) on a StepOnePlus system (Applied Biosystems, Waltham, MA, USA). Relative mRNA levels were calculated by the 2^-ΔΔCt^ method with β-actin as the internal control. Primer sequences are listed in Supplementary Table S3.

## 19. Targeted Energy Metabolomics Analysis

## Targeted quantification of energy metabolism-related metabolites was performed by Metabo-Profile Biotechnology (Shanghai, China) using an ultra-performance liquid chromatography-tandem mass spectrometry (UPLC-MS/MS) system (Acquity I-Xevo TQ-S, Waters Corp., Milford, MA, USA). Briefly, cell samples were thawed on ice to prevent metabolite degradation, and ultrasonicated in pre-chilled 80% methanol supplemented with internal standards. After centrifugation at 18,000 g for 15 min at 4 °C, supernatants were collected and derivatized with 3-nitrophenylhydrazine (3-NPH) and 1-ethyl-3-(3-dimethylaminopropyl) carbodiimide (EDC) at 30 °C for 60 min. The reaction was quenched by adding ice-cold methanol, and the mixture was centrifuged at 4,000 g for 20 min at 4 °C prior to injection.

## Chromatographic separation was performed on a BEH C18 analytical column (1.7 μm, 2.1 × 100 mm) maintained at 40 °C. The mobile phase consisted of 5 mM DIPEA aqueous solution (phase A) and acetonitrile/isopropanol (7:3, v/v, phase B), delivered at a flow rate of 0.3 mL/min with a gradient elution program. Mass spectrometric detection was operated in negative electrospray ionization (ESI⁻) mode with optimized source parameters. Pooled quality control (QC) samples (mixed from all experimental samples) and reagent blanks were inserted every 14 test samples throughout the analytical run to monitor system stability, and all samples were analyzed in random order. Raw data were processed with MassLynx v4.1 software (Waters) for peak integration and absolute quantification *via* external standard curves, and downstream statistical analysis was performed using the iMAP v1.0 platform (Metabo-Profile).

## 20. FBP1 Enzymatic Activity Assay

## The enzymatic activity of fructose-1,6-bisphosphatase 1 (FBP1) was measured using a commercial FBP activity assay kit (Solarbio, Beijing, China; Cat. No. BC0925) based on the coupled enzymatic method, following the manufacturer’s instructions. Briefly, cells were harvested and ultrasonicated in pre-chilled extraction buffer on ice, and the supernatant was collected after centrifugation at 8000 × g for 10 min at 4 °C. The reaction system was incubated at 37 °C, and the absorbance at 340 nm was detected at 10 s and 310 s respectively to calculate the generation rate of NADPH. FBP1 activity was normalized to total protein concentration; one unit of enzyme activity was defined as the amount of enzyme that produces 1 nmol of NADPH per minute per milligram of total protein.

## 21. Glycolytic Activity Assays

### Glucose Consumption: Measured using a Glucose Content Assay Kit (Solarbio; Cat. No. BC2500). Supernatants were collected after 24 h serum-free culture, and glucose content was detected at 505 nm. Lactate Production: Measured using a Lactic Acid Assay Kit (Solarbio; Cat. No. BC2210). Lactate concentration was detected at 530 nm and normalized to cell number. Maximal Glycolytic Capacity: Cells were incubated with 1.25 μM oligomycin (MCE; Cat. No. HY-N6782) for 3 h. Lactate was measured with a Glycolysis Assay Kit (Dojindo, Kumamoto, Japan; Cat. No. G270) at 450 nm. Maximal glycolytic capacity was calculated as nmol lactate/(10⁶ cells·h).

## 22. PKM Enzyme Activity Assay

PKM activity was measured using a Pyruvate Kinase Activity Assay Kit (Solarbio; Cat. No. BC0545) based on the coupled enzymatic method. The kit reaction system contains all essential cofactors and substrates for pyruvate kinase catalytic function, including phosphoenolpyruvate (PEP), ADP, MgCl₂, KCl, NADH, and lactate dehydrogenase as the coupling enzyme.

For in vitro recombinant protein assays, 1 μg purified GST-PKM2 was pre-incubated with equal molar amounts of His-FBP1 G260R or His-FBP1 G260R-Δ5 in kit reaction buffer overnight at 4 °C, followed by activity detection according to the manufacturer’s protocol. For cellular assays, THLE-2 cell lysates were used for activity measurement. Enzyme activity was normalized to total protein concentration.

**23. Serum Insulin Detection**

Serum insulin concentrations were measured using a mouse double-antibody sandwich enzyme-linked immunosorbent assay (ELISA) kit (Solarbio, Cat. No. SEKM-0387) following the manufacturer’s instructions. Briefly, serum samples were diluted appropriately to ensure insulin concentrations fell within the quantitative range of the standard curve. After termination of the chromogenic reaction, absorbance at 450 nm was detected with a microplate reader. The insulin concentration of each sample was calculated *via* four-parameter curve fitting based on the standard curve.

## **24. Hepatic Glycogen Content Measurement**

## Hepatic glycogen content was detected using a glycogen content assay kit (Solarbio, Cat. No. BC0345) based on the anthrone colorimetric method, following the manufacturer’s instructions. Briefly, weighed frozen liver tissues were homogenized and boiled in alkaline extraction solution to fully extract glycogen. After centrifugation to remove impurities, the supernatant was collected and reacted with anthrone working solution in a boiling water bath under strong acidic conditions. The absorbance value was measured at 620 nm with a microplate reader. Glycogen concentration was calculated according to the glucose standard curve, converted by the coefficient 1.11 (glucose to glycogen), and finally normalized to the weight of fresh liver tissue.

## **25.** Molecular Docking

## Protein structures of FBP1 (Uniprot ID: P09467, PDB ID: 2JJK) and PKM2 (Uniprot ID: P14618, PDB ID: 3G2G) were retrieved from the Protein Data Bank and preprocessed using PyMOL 2.5.5. Molecular docking was performed using the HADDOCK 2.4 server. Initial docking models were generated, followed by simulated annealing and molecular dynamics optimization. The best-scoring complex was selected based on the HADDOCK score, cluster size, and RMSD values. Protein-protein interaction interfaces and 2D interaction diagrams were analyzed and visualized using PyMOL 2.5.5 and LigPlot+ 2.2.8, respectively.

**26. *In Situ* Lactate Fluorescence Imaging**

For in situ detection of intracellular lactate distribution, AML12 cells transfected with eGFP-FBP1 were incubated with 100 μM PS-SCOTfluor-12 (MCE; Cat. No. HY-B2227) in pre-warmed complete culture medium containing serum for 2 h at 37 °C in the dark. After incubation, cells were washed three times with warm PBS to remove unbound probe, and then imaged by confocal laser scanning microscopy. Green fluorescence corresponds to eGFP-tagged FBP1, and red fluorescence represents the intracellular lactate signal.

## 27. Statistical Analysis

Statistical analysis was performed using SPSS 26.0, R 4.3.0, and GraphPad Prism 9. Normally distributed data are presented as mean ± standard deviation (SD) or mean ± standard error of the mean (SEM); non-normally distributed data as median (interquartile range, IQR). Categorical data are shown as n (%). For comparisons between two groups, two-tailed unpaired Student’s t-test was used for normally distributed variables, and the Mann-Whitney U test (Wilcoxon rank-sum test) was used for non-normally distributed variables. For comparisons among multiple groups, one-way ANOVA followed by Tukey’s test was used for normally distributed variables, and the Kruskal-Wallis test followed by Dunn’s test was used for non-normally distributed variables. Categorical variables were compared by χ² test. Propensity score matching was performed using the nearest-neighbor matching algorithm with a caliper width of 0.2. *p* values < 0.05 were considered statistically significant.

## 28. Supplementary Materials

All supplementary materials are available with the online version of this article. Supplementary Table S1 shows the detailed clinical and histological characteristics of enrolled patients. Supplementary Table S2 lists the primary and secondary antibodies used in this study. Supplementary Table S3 provides the primer sequences for quantitative real-time PCR.

**Supplementary figures**

**Figure S1. Establishment and validation of three diet-induced MASLD mouse models.** (A) Body weight curves of C57BL/6 male mice fed CD, CDAHFD (6 w), HFHCD (28 w) or WD (24 w). (B) Liver-to-body weight ratios at MASL (CDAHFD 4 w, HFHCD 12 w, WD 14 w) and MASH (CDAHFD 6 w, HFHCD 28 w, WD 24 w) stages. (C) Gross morphological appearance of liver tissues from each group. (D-E) Histopathological analysis: H&E (steatosis), Sirius Red (fibrosis) and Oil Red O (neutral lipid) staining of CDAHFD (D), HFHCD and WD (E) livers. Scale bars: 100 μm. (F) qPCR analysis of *Hk2*, *Pkm2*, *Pklr* and *Eno1* mRNA in CD and HFHCD livers at 12w (MASL) and 28 w (MASH). (G) Transcriptomic analysis of *FBP1* mRNA levels (nTPM) in healthy and MASH patient livers. (H) Immunoblot analysis of total Hnf4α protein in liver tissues from mice at different MASLD stages. Mice were fed a control diet or high-fat high-cholesterol diet for 12 or 28 weeks respectively, and β-actin served as the loading control. (I) Immunoblot detection of Hnf4α in cytosolic and nuclear fractions of mouse liver tissues. Hsp90 was used as the cytosolic marker and Lamin B1 as the nuclear marker to verify the purity of subcellular fractionation. For Panel A: Two-way ANOVA was performed. For Panels B, F and G: unpaired Student’s t-test was used compared with the control group. All experiments included 6 biological replicates (independent mice) per group (*n* = 6). Data are presented as mean ± SEM. * *p* < 0.05, ** *p* < 0.01, *** *p* < 0.001, **** *p* < 0.0001.

**Figure S2. MS/MS spectra validation of key FBP1-interacting glycolytic enzymes.** (A-C) Representative MS/MS spectra of unique peptides derived from PKM2 (A), ENO1 (B) and LDHA (C).

**Figure S3. Biophysical characterization of FBP1-mediated glycolytic compartmentalized complexes.** (A) Co-IP confirming interaction between HA-TurboID-FBP1 and endogenous PKM2/ENO1 in THLE-2 cells. β-tubulin served as loading control. (B) WB analysis of eGFP-FBP1 expression in HEK293 cells transfected with 1, 2 or 4 μg plasmid. β-Actin served as loading control. (C) Quantitative analysis of FBP1 puncta area fraction in HEK293 and AML12 cells transfected with increasing amounts of eGFP-FBP1. The puncta area fraction was defined as the percentage of FBP1-positive punctate area relative to the total cytoplasmic area, with three independent replicates (*n* = 3 per group). (D) Immunofluorescence showing dose-dependent formation of eGFP-FBP1 punctate compartmentalized complexes in HepG2 cells (3 μg vs 6 μg plasmid). Images are representative of three independent biological replicates. Scale bars: 5 μm. (E) Endogenous Fbp1 immunofluorescence in normal and MASH mouse liver tissues. Green: Fbp1; blue: DAPI (nuclei). Scale bars: 5 μm. (F) Endogenous Fbp1 immunofluorescence in AML12 normal mouse hepatocytes. Green: Fbp1; blue: DAPI (nuclei). Scale bars: 5 μm. (G) WB analysis of mCherry-PKM2 expression in HEK293 cells transfected with increasing amounts of plasmid. β-Actin served as loading control. (H) Subcellular localization of mCherry-PKM2 at different expression levels in HEK293 cells. Scale bars: 5 μm. (I) FRAP assay of eGFP-FBP1 condensates in HEK293 cells. Representative images and quantitative fluorescence recovery kinetics. Scale bars: 5 μm. (J) Effect of 1.5% 1,6-hexanediol on eGFP-FBP1 compartmentalized complexes, imaged at 0, 5, 10 and 15 min post-treatment. Scale bars: 5 μm. (K) Nuclear-cytoplasmic fractionation assay in AML12 cells transfected with empty GFP vector or GFP-FBP1, with duplicate technical loadings per condition. Both high- and low-exposure blots of PKM2 are presented. GAPDH is used as the cytosolic fraction marker and Lamin B1 as the nuclear fraction marker to validate fraction purity.

Data are presented as mean ± SEM. Significance was assessed by one-way ANOVA followed by Tukey’s test. ** *p* < 0.01, *** *p* < 0.001.

**Figure S4. Molecular docking analysis of the FBP1-PKM2 interaction interface.** (A) Overall structure of the FBP1-PKM2 complex (FBP1: cyan, PKM2: magenta, cartoon representation). (B) Magnified view of the binding interface, with interacting residues shown as sticks. Blue lines: hydrogen bonds; red lines: salt bridges.

**Figure S5. Validation of FBP1 mutant properties and functional assay controls.** (A) FBP1 enzymatic activity assay validating the catalytic inactivation effect of the G260R mutant. Left panel: total enzyme activity; right panel: specific activity corrected for protein expression levels (*n* = 6 per group). (B) Co-IP analysis of the interaction between HA-FBP1 G260R and endogenous PKM2 in HEK293T cells. (C) Subcellular localization of eGFP-tagged WT FBP1 and FBP1 G260R in HEK293 cells. Scale bars: 5 μm. (D) Colocalization of eGFP-FBP1 (WT/G260R) and mCherry-PKM2 in HEK293 cells. Scale bars: 5 μm. (E) Heatmap analysis of all detected glycolysis-related metabolites. (F-L) Quantitative gray value analysis of endogenous co-immunoprecipitation results, showing dynamic binding intensity between Fbp1 and glycolytic enzymes (Pkm2, Eno1, Ldha) in liver tissues from CD, 12-week HFHCD (MASL) and 28-week HFHCD (MASH) mice (*n* = 3 per group). (M) WB analysis of protein expression in THLE-2 cells transfected with Flag-PKM2 alone, or co-transfected with Flag-PKM2 and HA-FBP1 G260R / HA-FBP1 G260R-Δ5. (N) Co-immunoprecipitation assay in HEK293T cells transfected with empty vector (–), HA-tagged wild-type FBP1, or HA-tagged FBP1 Δ189-235 mutant. Immunoprecipitation was performed using an anti-HA antibody, and co-precipitated endogenous ENO1 and LDHA were detected by Western blot. (O, P) Relative glucose consumption (O) and relative lactate production (P) in THLE-2 cells co-transfected with Flag-PKM2 together with empty vector, HA-FBP1 G260R, HA-FBP1 G260R-Δ5 (binding-deficient mutant), or HA-FBP1 G260R-Δ7 (compartmentalization-deficient mutant). (Q) WB validation of Fbp1 knockdown and mutant reconstitution efficiency in AML12 hepatocytes. (R) BODIPY staining of neutral lipids in Fbp1-knockdown AML12 cells reconstituted with different FBP1 mutants. Scale bars: 5 μm. (S) BODIPY staining of neutral lipids in HepG2 cells transfected with different FBP1 mutants, validating the conserved lipid-regulating effect. Scale bars: 20 μm.

α-Tubulin or β-actin served as the loading control for all WB assays. Nuclei were stained with DAPI for all immunofluorescence assays. Data are presented as mean ± SEM. Significance was assessed by one-way ANOVA followed by Tukey's test. * *p* < 0.05, ** *p* < 0.01, *** *p* < 0.001, **** *p* < 0.0001 vs. control group.

**Figure S6. Validation of AAV8-mediated hepatic gene expression, fibrosis markers and gluconeogenic metabolic parameters.** (A) Schematic of liver-specific AAV8 constructs encoding HA-FBP1 G260R and HA-FBP1 G260R-Δ5 (189–235 aa deletion). ITR: inverted terminal repeat; Tbg: thyroxine-binding globulin promoter. (B) WB validation of HA-FBP1 variant expression in mouse liver tissues at 3 weeks after AAV8 tail vein injection. (C) Quantitative analysis of α-SMA protein levels in liver tissues from CDAHFD (4 w) or HFHCD (12 w) mice (*n* = 3 per group). (D) Hepatic glycogen content in HFHCD-fed MASLD mice reconstituted with FBP1 G260R or G260R-Δ5. (E) Fasting blood glucose levels in HFHCD-fed MASLD mice with the above treatments. (F) Serum insulin concentrations in HFHCD-fed MASLD mice with the above treatments.

β-actin served as the loading control for all WB assays. Data are presented as mean ± SEM. Significance was assessed by one-way ANOVA followed by Tukey's test for panel C, and two-sided unpaired Student's t-test for panels D-F. *n* = 6 per group for metabolic parameter assays. * *p* < 0.05, ** *p* < 0.01, *** *p* < 0.001, **** *p* < 0.0001.

**Supplementary tables**

**Table S1. Baseline clinical characteristics of patients with MASL and MASH**

| **Characteristic** | **MASL Group (*n* = 10)** | **MASH Group (*n* = 10)** | ***p*- value** |
| --- | --- | --- | --- |
| Sex, male/female | 5/5 | 5/5 | 1.000 |
| Age, years | 25.0 ± 8.2 | 32.5 ± 9.1 | 0.062 |
| BMI, kg/m² | 41.91 ± 8.76 | 37.34 ± 7.23 | 0.215 |
| Hypertension, n (%) | 3 (30.0) | 2 (20.0) | 0.628 |
| Type 2 diabetes mellitus, n (%) | 1 (10.0) | 5 (50.0) | 0.057 |
| Fasting glucose, mmol/L | 4.76 ± 0.72 | 6.27 ± 2.15 | 0.051 |
| HbA1c, % | 5.51 ± 0.42 | 6.53 ± 1.42 | 0.048* |
| Fasting insulin, mIU/L | 196.8 ± 132.5 | 226.0 ± 98.7 | 0.587 |
| ALT, U/L | 34.8 ± 17.2 | 76.3 ± 48.2 | 0.019* |
| AST, U/L | 28.7 ± 8.9 | 46.5 ± 19.8 | 0.018* |
| ALT/AST ratio | 1.18 ± 0.32 | 1.55 ± 0.57 | 0.089 |
| ALP, U/L | 81.6 ± 25.4 | 82.4 ± 19.7 | 0.937 |
| GGT, U/L | 33.7 ± 18.5 | 55.5 ± 20.6 | 0.022* |
| Steatosis percentage, % | 26.0 ± 21.7 | 67.0 ± 14.2 | < 0.001*** |
| NAS score | 1.5 ± 0.7 | 4.4 ± 1.3 | < 0.001*** |
| SAF activity score (A) | 0.2 ± 0.4 | 1.7 ± 1.1 | < 0.001*** |
| Fibrosis stage ≥F1, n (%) | 7 (70.0) | 10 (100.0) | 0.070 |

Note: Data are presented as mean ± standard deviation for normally distributed continuous variables, median (interquartile range) for non-normally distributed continuous variables, or number (percentage) for categorical variables. Group comparisons were performed using independent samples t-test (normal distribution), Mann-Whitney U test (non-normal distribution), or χ² test (categorical variables). For detailed statistical methods, check out the Materials and Methods Statistical Analysis' * *p* < 0.05, ** *p* < 0.01, *** *p* < 0.001. Abbreviations: MASL = metabolic dysfunction-associated steatotic liver; MASH = metabolic dysfunction-associated steatohepatitis; BMI = body mass index; HbA1c = glycated hemoglobin; ALT = alanine aminotransferase; AST = aspartate aminotransferase; ALP = alkaline phosphatase; GGT = gamma-glutamyl transferase; NAS = NAFLD Activity Score; SAF = steatosis, activity, fibrosis.

**Table S2. Antibodies used in this study**

All antibodies were used at the dilutions recommended by the respective manufacturers.

| **Antibody name** | **Target  protein** | **Host species** | **Isotype** | **Manufacturer (City, Country)** | **Catalog number** |
| --- | --- | --- | --- | --- | --- |
| Primary antibodies |  |  |  |  |  |
| HRP-conjugated Streptavidin | Biotin | - | - | Proteintech (Wuhan, China) | SA00001-0 |
| Anti-FBP1 | FBP1 | Rabbit | Polyclonal IgG | Proteintech (Wuhan, China) | 12842-1-AP |
| Anti-FBP1 | FBP1 | Mouse | Monoclonal IgG | Proteintech (Wuhan, China) | 68446-1-lg |
| Anti-PKM2 | PKM2 | Rabbit | Polyclonal IgG | Proteintech (Wuhan, China) | 15822-1-AP |
| Anti-ENO1 | ENO1 | Rabbit | Polyclonal IgG | Proteintech (Wuhan, China) | 11204-1-AP |
| Anti-LDHA | LDHA | Rabbit | Polyclonal IgG | Proteintech (Wuhan, China) | 19987-1-AP |
| Anti-HA tag | HA | Mouse | Monoclonal IgG | Proteintech (Wuhan, China) | 51064-2-IG |
| Anti-Flag tag | Flag | Rabbit | Polyclonal IgG | Proteintech (Wuhan, China) | 20543-1-AP |
| Anti-GFP tag | GFP | Rabbit | Polyclonal IgG | Proteintech (Wuhan, China) | 50430-2-AP |
| Anti-mCherry tag | mCherry | Rabbit | Polyclonal IgG | Proteintech (Wuhan, China) | 24675-1-AP |
| Anti-His tag | His | Rabbit | Polyclonal IgG | Proteintech (Wuhan, China) | 10001-0-AP |
| Anti-α-SMA | α-Smooth muscle actin | Rabbit | Polyclonal IgG | Proteintech (Wuhan, China) | 14395-1-AP |
| Anti-β-actin | β-Actin | Mouse | Monoclonal IgG | Proteintech (Wuhan, China) | 66009-1-IG |
| Anti-α-Tubulin | α-Tubulin | Rabbit | Polyclonal IgG | Proteintech (Wuhan, China) | 11224-1-AP |
| Secondary antibodies |  |  |  |  |  |
| HRP-conjugated goat anti-mouse IgG | Mouse IgG | Goat | Polyclonal IgG | Jackson ImmunoResearch (West Grove, PA, USA) | 115-035-003 |
| HRP-conjugated goat anti-rabbit IgG | Rabbit IgG | Goat | Polyclonal IgG | Jackson ImmunoResearch (West Grove, PA, USA) | 111-035-003 |
| HRP-conjugated anti-rabbit IgG (conformation-specific) | Rabbit IgG | Goat | Polyclonal IgG | Vazyme (Nanjing, China) | A0208 |
| HRP-conjugated anti-mouse IgG (conformation-specific) | Mouse IgG | Goat | Polyclonal IgG | Vazyme (Nanjing, China) | A0209 |

**Table S3. Primer sequences used for qPCR**

All primers were designed for Mus musculus genes and synthesized by GenScript Biotech Corporation (Nanjing, China).

| **Gene symbol** | **Forward primer (5'→3')** | **Reverse primer (5'→3')** |
| --- | --- | --- |
| *Fbp1* | CACCGCGATCAAAGCCATCT | AGGTAGCGTAGGACGACTTCA |
| *Pkm2* | GCCGCCTGGACATTGACTC | CCATGAGAGAAATTCAGCCGAG |
| *Eno1* | TGCGTCCACTGGCATCTAC | CAGAGCAGGCGCAATAGTTTTA |
| *Ldha* | TGTCTCCAGCAAAGACTACTGT | GACTGTACTTGACAATGTTGGGA |
| *Hk2* | TGATCGCCTGCTTATTCACGG | AACCGCCTAGAAATCTCCAGA |
| *Pklr* | TCAAGGCAGGGATGAACATTG | CACGGGTCTGTAGCTGAGTG |
| *Tgfb1* | ATTTGGAGCCTGGACACACA | GAGCGCACAATCATGTTGGA |
| *Col1a1* | TGCTAACGTGGTTCGTGACCGT | ACATCTTGAGGTCGCGGCATGT |
| *Col1a2* | TTGCTGAGGGCAACAGCAGGTT | AATGTCAAGGAACGGCAGGCGA |
| *Col3a1* | ACGTAAGCACTGGTGGACAG | CCGGCTGGAAAGAAGTCTGA |
| *Acta2* | CCCAGACATCAGGGAGTAATGG | TCTATCGGATACTTCAGCGTCA |
| *Timp1* | GAGACCACCTTATACCAGCGTT | TACGCCAGGGAACCAAGAAG |
| *Tnfa* | CATCTTCTCAAAATTCGAGTGACAA | TGGGAGTAGACAAGGTACAACCC |
| *Il1b* | GCAACTGTTCCTGAACTCAACT | ATCTTTTGGGGTCCGTCAACT |
| *Ccl2* | TACAAGAGGATCACCAGCAGC | ACCTTAGGGCAGATGCAGTT |
| *Il6* | TAGTCCTTCCTACCCCAATTTCC | TTGGTCCTTAGCCACTCCTTC |
| *Actb* | GTGACGTTGACATCCGTAAAGA | GCCGGACTCATCGTACTCC |

**Supplementary references**

1.	EASL-EASD-EASO Clinical Practice Guidelines on the Management of Metabolic Dysfunction-Associated Steatotic Liver Disease (MASLD). Obesity facts. 2024;17(4):374-444.

2.	Kleiner DE, Brunt EM, Van Natta M, Behling C, Contos MJ, Cummings OW, et al. Design and validation of a histological scoring system for nonalcoholic fatty liver disease. Hepatology. 2005;41(6):1313-21.

3.	Bedossa P, Poitou C, Veyrie N, Bouillot JL, Basdevant A, Paradis V, et al. Histopathological algorithm and scoring system for evaluation of liver lesions in morbidly obese patients. Hepatology. 2012;56(5):1751-9.
